# Bridging Psychological Stress and Skin Cellular Aging: Flavonoids as a Dual‐Action Therapeutic Strategy

**DOI:** 10.1002/ptr.70239

**Published:** 2026-02-04

**Authors:** Marco Duarte, Sílvia Santos Pedrosa, P. Raaj Khusial, Ana Raquel Madureira

**Affiliations:** ^1^ Universidade Católica Portuguesa, CBQF—Centro de Biotecnologia e Química Fina—Laboratório Associado, Escola Superior de Biotecnologia Porto Portugal; ^2^ Biorbis, Unipessoal LDA, Edifício de Biotecnologia da Universidade Católica Portuguesa Porto Portugal; ^3^ Independent Researcher Highland Mills New York USA

**Keywords:** flavonoids, hallmarks of aging, psychological stress, skin, stress mediators

## Abstract

Psychological stress (or simply “stress”) is a major contributor to chronic disease worldwide, affecting 35% of the global population, including younger generations. Furthermore, it plays a significant role in human premature aging; hence, its detrimental effects on people's health compel us to comprehend and control the ways in which psychological stress impacts our bodies, including our skin. For example, flavonoids, a class of polyphenolic phytochemicals, are an important group of plant secondary metabolites and appear as a promising solution. These compounds exhibit a number of general biological activities, such as anti‐inflammatory and antioxidant properties, as well as certain skin‐specific ones, like wound healing, photoprotection, and the treatment of inflammatory and cancerous disorders associated with the skin. For this reason alone, flavonoids could be regarded as a promising solution. Further, these substances have demonstrated beneficial effects on the different hallmarks of aging, demonstrating their potential as anti‐aging agents. They also have the ability to influence hormones linked to stress, which, considering their effects on skin health and aging mechanisms, seems to suggest that flavonoids may be effective ways to mitigate the negative effects of stress on premature skin aging. Therefore, this review seeks to demonstrate the potential of flavonoids as potential anti‐aging agents for the skin, either by improving the so‐called hallmarks of aging or by directly protecting the skin from external aggressors like UV radiation while reducing the negative effects of psychological stress and its known mediators.

**Abbreviations**
8‐OHdG: 8‐hydroxy‐2′‐deoxyguanosine
AD: Alzheimer's disease
Akt/PKB: protein kinase B
ALP: alkaline phosphatase
AMPK: AMP‐activated protein kinase
ATG: autophagy‐related protein
Bax: Bcl‐2‐associated X protein
Bcl‐2: B‐cell lymphoma 2
BMP2: bone morphogenetic protein 2
COX: cyclooxygenase
CXCL: chemokine (C‐X‐C motif) ligand
EGCG: epigallocatechin‐3‐gallate
ERK: extracellular signal‐regulated kinase
H_2_O_2_
: hydrogen peroxide
HPA: hypothalamic–pituitary–adrenal
IL: interleukin
iNOS: inducible nitric oxide synthase
JNK: c‐Jun N‐terminal kinase
LC3: microtubule‐associated protein light chain 3
MAPK: mitogen‐activated protein kinases
MIC: minimum inhibitory concentration
MMP: matrix metalloproteinase
MRSA: methicillin‐resistant *Staphylococcus aureus*

mTOR: mammalian target of rapamycin
NLRP3: NOD‐, LRR‐ and pyrin domain‐containing protein 3
OCN: osteocalcin
PGE2: prostaglandin E_2_
pSTAT: phosphorylated signal transducer and activator of transcription
ROS: reactive oxygen species
Runx2: runt‐related transcription factor 2
SASP: senescence‐associated secretory phenotype
SA‐*β*‐gal: senescence‐associated *β*‐galactosidase
TGF: transforming growth factor
TNF: tumor necrosis factor

## 1 Introduction

The skin is the largest human organ (including the hypodermis) and has numerous functions, such as protecting against trauma, solar radiation, toxins, and infections, preserving water and electrolytes, regulating temperature, and storing water, vitamin D, and fat. Moreover, the skin plays a role in blood pressure regulation and excretory physiological functions (Castro et al. 2023; Gilaberte et al. 2016; Walters and Roberts 2002). The skin suffers changes throughout time, progressively losing its ability to adapt to its surroundings, by a process we know as “aging”. This process is characterized by a decrease in structural integrity and physiological function, and it is mainly caused by extrinsic (environmental) factors (e.g., UV radiation), but also by intrinsic (chronological) ones (Castro et al. 2023), being psychological stress an important accelerator of the aging process. For instance, Duarte et al. (2024) thoroughly describe the effects of stress and its mediators on skin physiology and cellular aging. Psychological stress is recognized as a major cause of long‐term illness worldwide, resulting in millions of workdays lost (O'Connor et al. 2021; Health and Safety Executive 2025). The repeated activation of stress responses, along with prolonged exposure to cortisol and other stress hormones, increases the risk of various health conditions, including anxiety, depression, heart disease, stroke, and sleep (American Psychological Association 2024). According to the American Institute of Stress, approximately 35% of the global population experiences stress, particularly younger generations facing heightened levels. Stress operates through neuroendocrine pathways, mainly by activating the hypothalamic–pituitary–adrenal (HPA) axis, culminating in the adrenal cortex with the release of glucocorticoids, notably cortisol, the so‐called “stress hormone” (O'Connor et al. 2021; The American Institute of Stress n.d.). In addition to the HPA axis and cortisol, catecholamines (epinephrine and norepinephrine) via adrenergic stimulation significantly contribute to the stress response (Radek 2010). Other minor stress mediators are, for example, Substance P, serotonin and melatonin (Duarte et al. 2024). Taking all this into consideration, it is safe to say that psychological stress affects the physical health of our bodies, with the skin being no exception (Lee, Watson, and Kleyn 2020).

**FIGURE 1:**
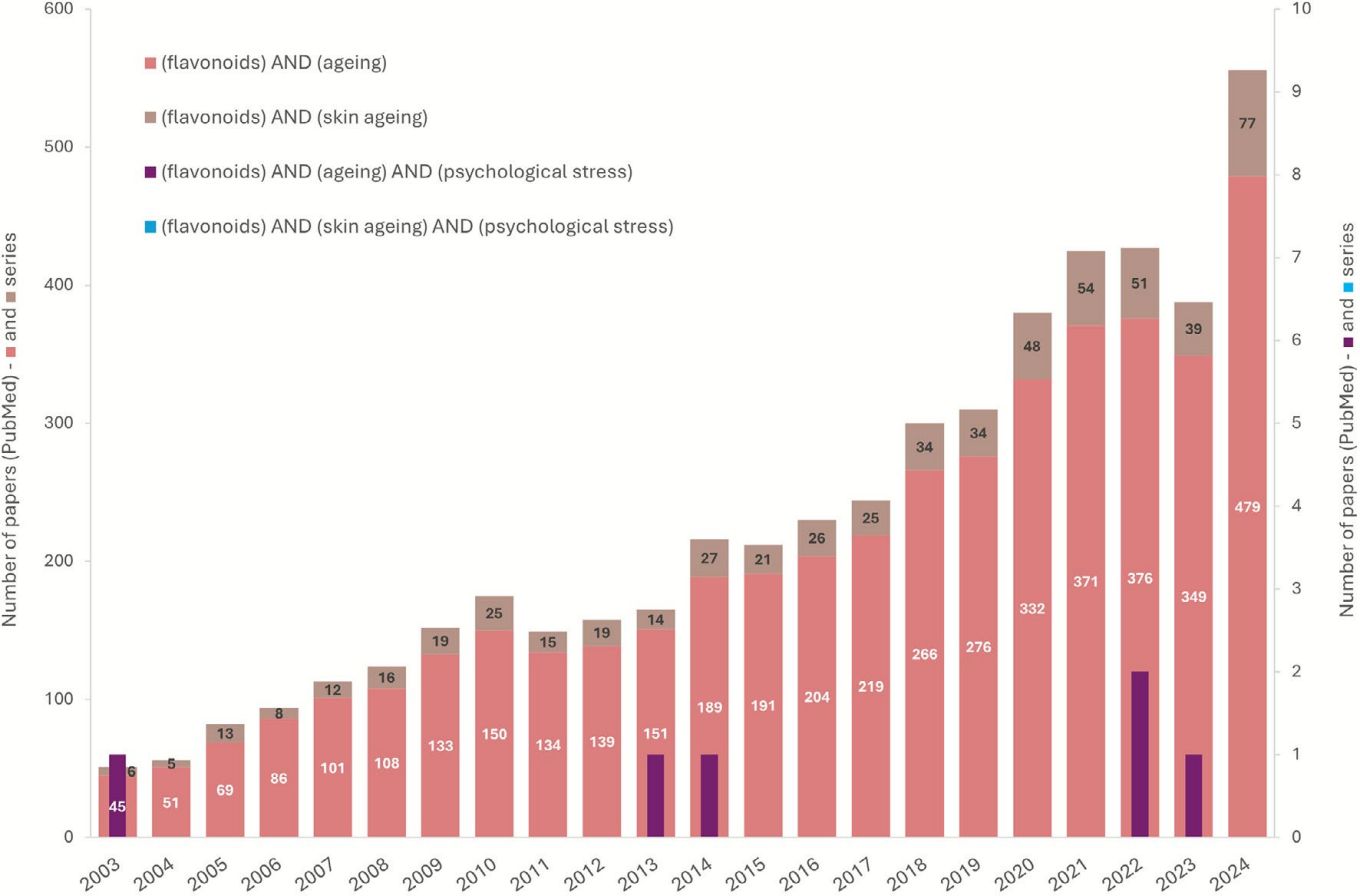
Number of papers in PubMed database, from 2003 to 2024, accordingly to the specified search strings. The scheme provides an overview of the amount of research conducted concerning the indicated topics over the years. The used search strings were: 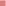 “(flavonoids) AND (aging)”; 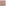 “(flavonoids) AND (skin aging)”; 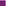 “(flavonoids) AND (aging) AND (psychological stress)”; 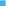 “(flavonoids) AND (skin aging) AND (psychological stress)”. The first two entries (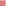 and 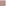 ) are relative to the left axis, while the last two (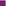 and 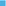 ) are relative to the right axis.

In line with this, a new interdisciplinary research field known as Geroscience has appeared recently, aiming to develop novel biologically‐driven therapeutic and preventive approaches to manage key aging mechanisms (Costa et al. 2025), which, importantly, have been reported to be modulated by psychological stress (Duarte et al. 2024). Indeed, nowadays there is a demand to understand how the skin‐related effects of psychological stress, especially aging, can be managed. Market forecasts report that the global anti‐aging cosmetics market is rapidly growing. For instance, according to Precedence Research, this market accounted for USD 56.71 billion in 2024, increased to USD 60.11 billion in 2025, and is predicted to be worth around USD 101.46 billion by 2034, with a solid CAGR of 5.99% between 2024 and 2034 (Precedence Research 2024). According to Fortune Business Insights, this growth is strictly related to the consumers' preferences. In fact, people examine not only the use and advantages of cosmetic items, but also the ingredients in them. Synthetic substances might cause adverse effects and allergic responses on the skin. Consumers' interest in non‐toxic anti‐aging cosmetic products has grown as they become more aware of the benefits of natural components. For example, according to a 2019 survey titled “Green Consumer Behaviour in the Cosmetics Market” conducted by Molecular Diversity Preservation International (MDPI), nearly 70% of 196 respondents in Hungary prefer to buy natural cosmetic products and are willing to pay more for this type of product (Fortune Business Insights 2025). For instance, a quick search through the website of Sephora Portugal is symptomatic of this. From 425 products categorized as “anti‐wrinkle and anti‐aging treatment”, nearly 31% (131) fall into the category of “responsible beauty,” which comprises products with certain sustainable features (e.g., vegan, refillable). In nature, we can find a vast selection of natural compounds that could be useful for these purposes. For instance, flavonoids are a major example. These are a class of polyphenolic phytochemicals, representing an important group of secondary metabolites in plants. In nature, they can be sourced from fruits, vegetables, herbs, stems, cereals, nuts, flowers, beverages, and seeds. To date, over 10,000 flavonoid compounds have been isolated and identified. In general, flavonoids consist of a 15‐carbon skeleton (C6–C3–C6) arranged in two benzene rings (C6; labelled A and B, Figure 1) and a 3‐carbon linking chain (C3, labelled C). Depending on their chemical structure, they can be divided into several subclasses, including flavonols, flavan‐3‐ols (flavanols or catechins), anthocyanidins, chalcones, flavones, isoflavones, and flavanones (Chen et al. 2023; Li, Qian, et al. 2023; Panche et al. 2016; Zulkefli et al. 2023). A review paper by our group thoroughly describes the biological potential of flavonoids, comprising activities such as antioxidant, anti‐inflammatory, antimicrobial, anticancer, antidiabetic, anti‐obesity, osteoprotective, hepatoprotective, neuroprotective, cardioprotective, gastrointestinal protection, and antinociceptive (Duarte et al. 2025).

The PubMed database was searched to find the number of papers published since 2003 that contained the following entry words: (1) “(flavonoids) AND (aging)”; (2) “(flavonoids) AND (skin aging)”; (3) “(flavonoids) AND (aging) AND (psychological stress)”; (4) “(flavonoids) AND (skin aging) AND (psychological stress)”. Figure 1 displays the search results. According to the findings, flavonoids' link to aging has drawn more attention recently. Yet, the number of papers is noticeably reduced when it comes to skin aging in particular. Importantly, it is clear that we are entering unknown terrain when we go further and include psychological stress in the equation, particularly if we limit the aging process to the skin. Actuality, any study was found comprising all three components, which is symptomatic of the novelty of the field. The year 2025 was left out due to its incompleteness.

In this paper, we aim to provide the readers with an insightful and complete review on the relationship of flavonoids with three separate topics: skin health, cellular aging and psychological stress. Nonetheless, our main objective is to highlight the gaps that exist in the exploration of these concepts (“flavonoids”, “skin aging”, “psychological stress”) together. As mentioned in the previous paragraph, no articles were found with the three words simultaneously and, therefore, it is crucial to make it clear that, from our perspective, this is the way to affirm flavonoids as real solutions to combat premature skin aging, in which psychological stress plays a prominent role.

## 2 Search Methodology

The search was carried out using Google Scholar and PubMed databases, with no time range restrictions. Nevertheless, the authors were careful to present the most recent cases possible. The search strings relied on cross‐referencing a flavonoid's name (e.g., apigenin, epicatechin, genistein) with the respective skin‐related topic (e.g., wound healing, melanoma), hallmark of aging (e.g., DNA damage, autophagy), or stress mediator (e.g., cortisol, epinephrine). For instance, “apigenin (AND) wound healing”, “kaempferol (AND) DNA damage”, or “genistein (AND) cortisol” could be examples of used search strings. Only studies exploring individual flavonoids, where the intended biological activity was clearly identified and studied, were considered. On the other hand, papers that exclusively reported the benefits of flavonoid‐enriched extracts were excluded from the search.

## 3 Psychological Stress, Skin Cellular Aging, and Flavonoids—Building a Connection

**FIGURE 2:**
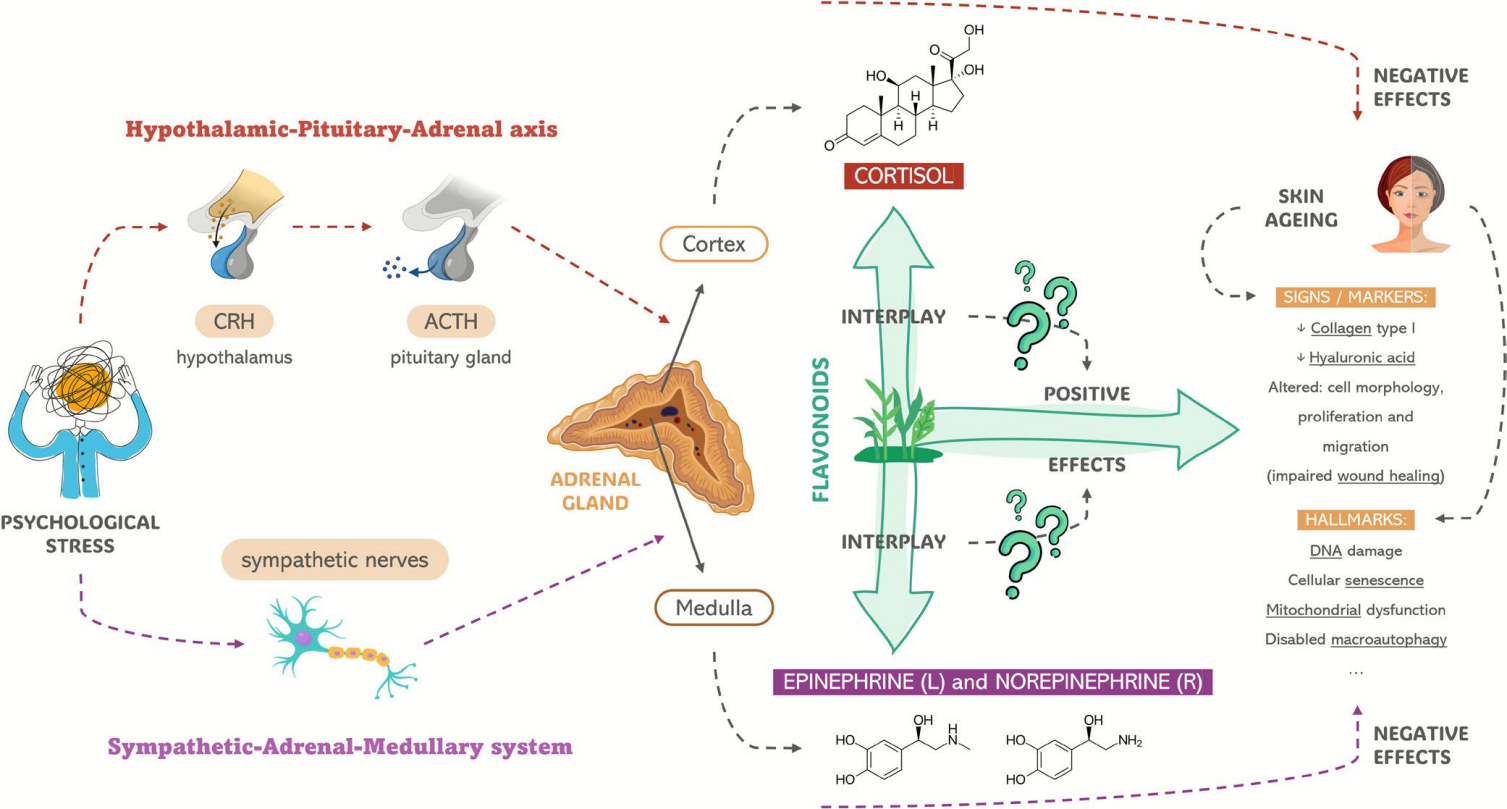
Schematic representation of the proposed relationship between flavonoids, psychological stress mediators, and skin aging.

As stated before, there is not a significant amount of research directly establishing a relationship between these topics. Despite the lack of studies, which is a strong indicator of the huge novelty of the field, we consider that the possibility for this connection to exist is promising and exciting, representing an innovative and disruptive development proposal. This review features research using different experimental models (humans, animals, in vitro), allowing us to examine how flavonoids affect skin health and biological aging systems, while simultaneously exploring their impact on neuroendocrine mediators of psychological stress (e.g., norepinephrine, epinephrine, cortisol, Substance P) that may contribute to these effects. At first sight, all these topics may seem disconnected. However, evidence has shown that psychological stress is transversely related both with skin health and the hallmarks of aging. For instance, a higher chance of developing age‐related diseases early in life and, more recently, an older biological age have been linked to exposure to long‐term stressful events and the subsequent activation of physiological responses, like the HPA axis. In fact, the impact of stress and/or its mediators over these molecular mechanisms has been studied and reported before, although mainly using tissue models other than skin (Duarte et al. 2024; Polsky et al. 2022). Further, the molecular significance of psychological stress in skin conditions such as atopic dermatitis and psoriasis has been studied, emphasizing the roles of cortisol levels in both diseases, corticotropin‐releasing hormone (CRH) and brain derived neurotrophic factor (BDNF) in psoriasis, and the HPA axis and interleukin (IL)‐18 in atopic dermatitis (Jamerson et al. 2022). While the former three are easily associated with neurobehavioral aspects, the relationship of IL‐18 with this topic is not as clear. Therefore, it is important to clarify the high relevance of this cytokine within this context given that it has been associated with psychiatric conditions such as depression and cognitive impairment. Also, IL‐18 deficiency might induce hippocampus damage, leading to depression‐like behavioral alterations. However, the potential significance of IL‐18 in stressful situations remains unclear (Yamanishi et al. 2022). To date, what we know is that flavonoids separately impact skin health, the hallmarks of aging, and psychological stress mediators, and that the latter can also impact the former two. However, from our point of view, the current knowledge on these topics falls short, as no evidence supports the possibility that flavonoids' downstream effects on skin health and the hallmarks of aging could be due to their prior upstream effects over the stress mediators. Pointing to highlight the importance of studying this relationship and developing models that allow us to do so, this review aims to open doors to future research pushing forward to advance the field, allowing us to “cut corners” and, therefore, establishing a direct link between flavonoids and stress‐induced skin cellular aging. A schematic illustration of the suggested link between flavonoids, psychological stress mediators, and skin molecular aging is shown in Figure 2, which provides a simple overview of how flavonoids may potentially prevent psychological stress‐induced skin aging.

### 3.1 Impact of Flavonoids on Skin Health

Flavonoids are found to exert specific health benefits on the skin, such as wound healing, photoprotection, skin cancer treatment, and common inflammatory diseases, among others. In fact, flavonoids are already used as active ingredients in many cosmetics, mostly for their antioxidant and soothing properties. Furthermore, the incorporation of flavonoid‐enriched extracts in skin care products is well‐known within the cosmetic industry for allowing topical formulations with skin‐protective qualities (Andrabi et al. 2024). Nonetheless, flavonoids' potential for cosmetic use is far from totally explored, as these compounds comprise a vast variety of relevant biological activities, including skin‐specific ones, as described in the next subsections and summarized in Table 1.

**TABLE 1:** Skin‐protective effects of flavonoids.

Activity	Flavonoid	Subclass	Model(s)	Outcomes	References
Wound healing					
	Luteolin	Flavone	In vivo streptozotocin‐induced impaired wound healing Wistar rat model	Ameliorated impaired healing and accelerated re‐epithelization of skin	(Chen et al. 2021)
	Quercetin	Flavonol	In vitro CCK‐8 and scratch assays; in vivo cutaneous wound C57BL/6 mouse model	Enhanced proliferation and migration of fibroblasts; improved wound healing capacity in vivo	(Mi et al. 2022)
	Naringin	Flavanone	In vivo biopsy punch‐induced wound C57BL/6 mouse model	Helped in the recovery of the wound site; activated HaCaT migration in vitro	(Yen et al. 2022)
	Genistein	Isoflavone	In vivo hemorrhagic shock‐induced impaired wound healing Sprague–Dawley rat model	Promoted skin wound healing by enabling the mobilization of endothelial progenitor cells to the wound site	(Peng et al. 2023)
	Puerarin	Isoflavone	In vitro dexamethasone‐treated HaCaT; in vivo dexamethasone‐induced impaired wound healing BALB/c mouse model	Promoted keratinocyte proliferation and migration; accelerated wound closure rate, increased capillaries density, and upregulated the level of collagen fibers and TGF‐β in vivo	(Nguyen et al. 2021)
	Epigallocatechin‐3‐gallate	Flavanol	In vivo skin wound Wistar rat model	Increase the efficiency of mesenchymal stem cells (MSC)‐induced skin wound closure; promoted a strong anti‐scarring effect during the healing stages	(Li et al. 2016)
	Phlorizin	Chalcone	In vivo burn wound ICR mouse model	Promoted re‐epithelialization and angiogenesis at the wound site	(Yang et al. 2024)
UV protection					
	Chrysin	Flavone	In vitro UVA‐ and UVB‐induced damage HaCaT cell model	Attenuated apoptosis, ROS production, and COX‐2 expression; reversed the downregulation of aquaporin 3 and decreased JNK and p38 activation	(Wu et al. 2011)
	Fisetin	Flavonol	In vivo UVB‐induced skin damage hairless mouse model	Reduced erythema and wrinkle development; decreased epidermal hyperplasia and increased collagen concentration in the dermis; boosted the expression of filaggrin, preventing barrier function impairment.	(Wu et al. 2017)
	Eriodictyol	Flavanone	In vitro UVA‐treated HaCaT cells	Reduced ROS production, while lowering MMP‐1 and inflammatory factors levels; upregulated TIMP‐1 and collagen‐1	(Nisar et al. 2022)
	Genistein	Isoflavone	In vivo UVB‐treated Sprague–Dawley rat model; in vivo human trial; in vitro UVB‐treated HaCaT cells	Reduced proinflammatory cytokines (CXCL1, IL‐1, MIF, PLANH1) in vitro; reduced wrinkles severity; ameliorated skin elasticity and hydration in humans	(Tang et al. 2022)
	Catechin	Flavanol	In vitro UVB‐induced damage HaCaT cell model	Prevented cellular death; inhibiting the generation of intracellular H_2_O_2_, and JNK activation	(Wu et al. 2006)
	Delphinidin	Anthocyanidin	In vitro UVB‐treated HaCaT cell model; in vivo UVB‐treated skin SKH‐1 hairless mouse model	Prevented decreased cell viability, cell apoptosis (↓ Bax, ↑ Bcl‐2) and lipid peroxidation, while decreasing DNA damage (↓ 8‐OHdG) in vitro; decreased apoptosis and DNA damage markers in vivo	(Afaq et al. 2007)
Skin cancer					
	Apigenin	Flavone	In vitro human melanoma A375SM and A375P cell lines; in vivo A375SM cells tumor xenograft BALB/c mouse model	Induced apoptosis of human melanoma cells (↑ Bax, ↓ Bcl‐2) in vitro; reduced tumor volume in vivo	(Woo et al. 2020)
	Kaempferol	Flavonol	In vitro human/mouse melanoma A375/B16F10 cells; in vivo A375 cell lung metastasis BALB/c mouse model	Inhibited migration and invasion of both cell lines; reduced lung metastasis in vivo	(Zheng et al. 2022)
	Naringenin	Flavanone	In vitro human epidermoid carcinoma (skin squamous‐cell carcinoma) A431 cell line	Reduced cell viability while increasing nuclear condensation and DNA fragmentation; increased ROS production and led to cell cycle arrest	(Ahamad et al. 2014)
	Genistein	Isoflavone	In vitro murine melanoma B16F10 cell line	Inhibited proliferation and induced apoptosis of melanoma cells	(Cui et al. 2017)
	Epigallocatechin‐3‐gallate	Flavanol	In vitro human epidermoid carcinoma A431 and SCC13 cell lines	Reduced cell viability and increased cell death (↓ β‐catenin signaling)	(Singh and Katiyar 2013)
	Delphinidin	Anthocyanidin	In vitro 12‐O‐tetradecanoylphorbol13‐acetate (TPA)–induced neoplastic mouse epidermal JB6 *P*+ cell transformation model	Significantly reduced neoplastic cell transformation in mouse epidermal JB6 *P*+ cells	(Kuo et al. 2019)
	Isoliquiritigenin	Chalcone	In vitro A375/A2058/B16 melanoma cell lines; in vivo melanoma lung metastasis C57BL/6 mouse model	Suppressed proliferation and migration of melanoma cells (↓ miR‐27a expression); inhibited the growth of B16‐based lung metastasis	(Xiang et al. 2021)
Skin Inflammatory Diseases					
Psoriasis	Apigenin	Flavone	In vivo imiquimod‐induced psoriasis C57BL/6J mouse model	Attenuated phenotypic changes, such as erythema, scaling and epidermal thickening; improved splenic hyperplasia	(Meng et al. 2024)
	Kaempferol	Flavonol	In vivo imiquimod‐induced psoriasis BALB/c mouse model	Ameliorated psoriasis‐like skin lesions; reduced dendritic and γδT17 cells populations; downregulated pro‐inflammatory cytokines (IL‐23, IL‐17A, TNF‐α, IL‐6, IL‐1β) and pathways (JAK–STAT)	(Li, Cui, et al. 2023)
	Pinocembrin	Flavanone	In vivo imiquimod‐induced psoriasis BALB/c mouse model	Improved skin psoriasis area and severity index score, epidermal thickness, inflammation, hyperplasia, hyperkeratosis, and CD4+ T‐cell infiltration; reduced inflammatory cytokines and keratinocyte proliferation markers	(Huang et al. 2022)
	Formononetin	Isoflavone	In vivo imiquimod‐induced psoriasis BALB/c mouse model	Improved psoriatic pathological features (erythema, scale, thickness of skin lesions); reduced IFN‐α, IFN‐β, IFN‐γ, TNF‐α and IL‐17 levels	(Xu et al. 2024)
	Delphinidin	Anthocyanidin	In vitro full‐thickness three‐dimensional reconstituted human psoriatic skin model	Decreased expression of psoriasis‐associated markers of proliferation (Ki67 and proliferating cell nuclear antigen) and inflammation (iNOS, S100A7‐psoriasin, S100A15‐koebnerisin); suppressed release of psoriasis‐associated keratinocyte‐derived proinflammatory cytokines	(Chamcheu et al. 2015)
Atopic dermatitis	Luteolin	Flavone	In vivo 2,4‐dinitrochlorbenzene‐induced atopic dermatitis BALB/c mouse model	Reduced IgE serum levels while improving injured skin tissue architecture; reduced oxidative stress, inflammation and white blood cells count	(Wang et al. 2021)
	Myricetin	Flavonol	In vivo dinitrofluorobenzene‐induced atopic dermatitis obese KM mouse model	Reduced serum IgE and histamine, inhibited the infiltration of CD 4+ T‐cells; modulated pro‐inflammatory factors expression; restored impaired barrier function	(Gao, Tang, et al. 2023)
	Hesperidin	Flavanone	In vivo atopic dermatitis NC/Nga mouse model	Reduced skin lesions and serum IgE level; reduced inflammatory markers (IL‐17, IFN‐γ)	(Nagashio et al. 2013)
	Epigallocatechin‐3‐gallate	Flavanol	In vivo *Dermatophagoides pteronissinus* ‐induced atopic dermatitis NC/Nga mouse model	Reduced total clinical severity score and ear thickness, while improving histological grading; reduced mRNA and protein levels of MIF, TNF‐α, and IFN‐γ	(Noh et al. 2008)
Acne vulgaris	Quercetin	Flavonol	In vitro *C. acnes*‐stimulated HaCaT, THP‐1 and RAW 264.7 cells; in vivo *C. acnes* intradermal injection BALB/c mouse model	Ameliorated inflammation in both cell lines; reversed some *C. acnes* ‐injection effects (↓ ear thickness, ↓ swelling) in vivo	(Lim et al. 2021)
	Phloretin	Chalcone	In vitro *C. acnes*‐stimulated HaCaT cells	Inhibited *C. acnes* growth; reduced *C. acnes*‐induced TLR 2‐mediated inflammation; reduced fatty acid synthesis, impairing bacterial survivability	(Cheon et al. 2019)
			In vitro C. acnes‐stimulated HaCaT cells; in vivo double‐blind placebo‐controlled study	Inhibited *C. acnes* growth and prevented bacterial‐induced inflammation (↓ COX‐2 promoter, ↓ PGE2) in vitro; reduced comedo counts and sebum output in vivo; decreased whiteheads, blackheads, papules, sebum secretion, and porphyrin levels in vivo	(Kum et al. 2016)
Others					
Skin fibrosis	Pinocembrin	Flavanone	In vitro proliferation, migration and invasion assays; in vivo bleomycin‐induced skin fibrosis C57BL/6 mouse model	Inhibited proliferation, migration, and invasion of keloid fibroblasts and mouse primary dermal fibroblasts in vitro; reduced gross weight and fibrosis‐related protein expression of keloid tissues in vivo	(Li, Zhai, et al. 2021)
Vitiligo	Apigenin	Flavone	In vivo hydroquinone‐induced vitiligo C57BL/6 mouse model	Reduced depigmentation, inflammatory markers, and oxidative stress, while increasing tyrosinase; increased melanin containing hair follicles	(Chauhan et al. 2024)

#### 3.1.1 Wound Healing

Flavonoids have shown relevant wound healing capacity through several mechanisms. For instance, quercetin and puerarin respectively improved the proliferation and migration of both fibroblasts and keratinocytes in vitro. In vivo research corroborates these effects, with flavonoids like luteolin, naringin, genistein, epigallocatechin‐3‐gallate (EGCG), or phlorizin being able to promote wound healing via improved re‐epithelization, angiogenesis, endothelial progenitor cells mobilization, or even increased collagen deposition at the wound sites. These findings highlight the potential of flavonoids as therapeutic agents in skin wounds.

Puerarin has been reported to promote keratinocyte proliferation and migration in dexamethasone‐treated HaCaT cells by activating the extracellular signal‐regulated kinases (ERK) and protein kinase B (Akt/PKB) pathways in vitro. Additionally, using an in vivo dexamethasone‐induced impaired wound healing BALB/c mouse model, topical application of puerarin allowed for the acceleration of wound closure rate, increased the density of the capillaries, and upregulated the level of collagen fibers and transforming growth factor (TGF)‐β in the wound sites (Nguyen et al. 2021). In another study, quercetin enhanced both the proliferation and migration of fibroblasts in vitro, as well as improved wound healing capacity in mice through the inhibition of inflammation and increase of growth factors expression (Mi et al. 2022). Moreover, using an in vivo streptozotocin‐induced impaired wound healing Wistar rat model, luteolin ameliorates impaired healing and accelerates skin re‐epithelization. Also, it decreased the expression of inflammatory factors (e.g., tumor necrosis factor [TNF]‐α, interleukin [IL]‐6) and increased antioxidant enzymes (e.g., superoxide dismutase [SOD], glutathione peroxidase [GSH‐Px]) (Chen et al. 2021). Furthermore, using an in vivo biopsy punch‐induced wound C57BL/6 mouse model, naringin was reported to help recover the wound site significantly. Also, it was found to activate HaCaT migration in vitro (Yen et al. 2022). In another study, Peng et al. (2023) reported that genistein was able to promote skin wound healing in an in vivo hemorrhagic shock‐induced impaired wound healing Sprague–Dawley rat model by enabling the mobilization of endothelial progenitor cells to the wound site through angiotensin II (Ang‐II), stromal cell‐derived factor‐1alpha (SDF‐1α), and TGF‐β signaling (Peng et al. 2023). In addition, EGCG was shown to increase the efficiency of mesenchymal stem cells (MSC)‐induced skin wound closure, as the flavonoid‐treated group presented the best results on several features such as epidermal thickness and neovascularization‐related growth factors expression while being associated with a strong anti‐scarring effect during the healing stages. Also, several pro‐inflammatory cytokines were downregulated, exacerbated by flavonoid treatment (Li et al. 2016). Lastly, phlorizin, loaded into chitosan nanofibers, was able to increase HaCaT viability in vitro while promoting re‐epithelialization and angiogenesis at the wound site and reducing the inflammatory response, using an in vivo burn wound ICR mouse model (Yang et al. 2024).

#### 3.1.2 Photoprotection

By controlling oxidative and inflammatory processes, as well as apoptosis and DNA damage, flavonoids demonstrate strong photoprotective effects. According to in vitro research, flavonoids shield skin cells from UV radiation by lowering pro‐inflammatory cytokines like cyclooxygenase (COX)‐2, IL‐1, and chemokine (C‐X‐C motif) ligand 1 (CXCL1) as well as oxidative markers like reactive oxygen species (ROS) and H_2_O_2_. These results are further supported by in vivo studies, showing that flavonoids improve skin structure (by boosting collagen and filaggrin) and features (wrinkles and hydration) when exposed to UV radiation. Additionally, certain flavonoids reduce DNA damage and cellular death caused by UV rays in both in vitro and in vivo models. These results imply that flavonoids may function as skin photoprotective agents.

Chrysin was shown to attenuate apoptosis, ROS production, and COX2 expression induced by both UVB and UVA in HaCaT cells. Also, it reversed UVB‐induced downregulation of aquaporin 3, reduced c‐Jun N‐terminal kinase (JNK) activation, and inhibited UVA‐ and UVB‐triggered p38 activation (Wu et al. 2011). Moreover, eriodictyol significantly reduced UVA‐mediated ROS production while lowering matrix metalloproteinase (MMP)‐1 and levels of inflammatory cytokines. Additionally, it upregulated tissue inhibitory metalloproteinase (TIMP)‐1 and collagen‐1. Similarly to chrysin, eriodictyol was also able to reduce JNK and p38, thus allowing the inhibition of MMP‐1 expression (Nisar et al. 2022). Furthermore, (+)‐catechin was reported to protect HaCaT cells from UVB‐induced death while inhibiting UVB‐mediated generation of intracellular hydrogen peroxide (H_2_O_2_) and JNK activation (Wu et al. 2006). In another study, genistein was found to reduce the UVB‐induced expression of proinflammatory cytokines (CXCL1, IL‐1, macrophage migration inhibitory factor (MIF), and plasminogen activator inhibitor‐1 (PLANH1)) in HaCaT cells. Using an in vivo UVB‐treated Sprague–Dawley rat model, it reduced the severity of UVB‐induced wrinkling. Also, in a 2‐year study conducted with human participants by the Department of Dermatology, Hualien Tzu Chi Hospital, Taiwan, skin features like elasticity and hydration were found to be improved by genistein (Tang et al. 2022). Furthermore, in vitro studies showed that delphinidin protected HaCaT cells against UVB‐induced decrease in cell viability, induction of apoptosis, increase in lipid peroxidation, formation of 8‐hydroxy‐20‐deoxyguanosine (8‐OHdG), decrease in proliferating cell nuclear antigen expression, increase in poly(ADP‐ribose) polymerase cleavage, activation of caspases, decrease in B‐cell lymphoma (Bcl)‐2, increase in Bcl‐2‐asspcoated X protein (Bax), upregulation of BH3 interacting domain death agonist (Bid) and Bcl‐2 homologous antagonist/killer 1 (Bak), and downregulation of Bcl‐xL (extra‐large). On the other hand, in vivo experiments in UVB‐treated SKH‐1 hairless mice showed that topical administration of delphinidin resulted in decreased apoptosis and DNA damage markers, such as cyclobutane pyrimidine dimers and 8‐OHdG (Afaq et al. 2007). Finally, using an in vivo UVB‐induced skin damage hairless mouse model, Wu et al.'s study results revealed that the topical application of fisetin reduced UVB‐induced erythema and wrinkle development. Furthermore, fisetin reduced epidermal hyperplasia and increased collagen concentration in the dermis. Also, it inhibited MMP‐1, MMP‐2, and COX‐2 expression while boosting nuclear factor erythroid 2‐related factor 2 (Nrf2) expression, resulting in photoprotection. Lastly, fisetin boosted the expression of filaggrin, preventing UVB‐induced barrier function impairment (Wu et al. 2017).

#### 3.1.3 Skin Cancer

Numerous authors have reported on flavonoids' preventive effect against several kinds of skin cancer. For instance, by increasing apoptosis and decreasing the ability of skin cells to migrate and invade in vitro, apigenin, kaempferol, genistein, and isoliquiritigenin prevented the growth of melanoma. This is supported by studies showing that flavonoids can lower the volume of melanoma tumors and lung metastases in vivo. Further, naringenin and EGCG decrease cell viability and increase cell death in human epidermoid carcinoma due to DNA fragmentation and oxidative stress. These findings highlight the potential of flavonoids as anti‐skin cancer agents.

Apigenin was shown to induce apoptosis of human melanoma cells (A375P and A375SM) by modulating the expression levels of apoptosis‐related proteins, decreasing Bcl‐2, and increasing Bax, cleaved poly ADP‐ribose polymerase, cleaved caspase‐9, and p53. In vivo experiments showed that apigenin was also able to reduce the A375SM‐based tumor volume in mice (Woo et al. 2020). Genistein significantly inhibited the proliferation and induced apoptosis of murine melanoma B16F10 cells while reducing p‐p38, p‐ERK, and p‐JNK levels, and the gene expression of focal adhesion kinase (FAK), paxillin, vimentin, and epithelial‐to‐mesenchymal transition‐related transcription factor SNAIL (Cui et al. 2017). Additionally, kaempferol was reported to inhibit migration and invasion of A375 and B16F10 cells in vitro and to reduce A375‐based lung metastasis in an in vivo mouse model. In this study, the effect of kaempferol on aerobic glycolysis of melanoma cells was explored, and the results revealed that kaempferol significantly reduced extracellular acidification rates (ECAR) and glucose intake, as well as the synthesis of ATP, pyruvate, and lactate. Also, it inhibited hexokinase, the first key kinase in aerobic glycolysis, and reduced the binding of hexokinase II and voltage‐dependent anion channel (VDAC) 1 to mitochondria via the AKT/glycogen synthase kinase (GSK)‐3β signal pathway. However, overall hexokinase II protein expression remained unchanged (Zheng et al. 2022). In another study, isoliquiritigenin was found to suppress the proliferation and migration of melanoma cells by downregulating miR‐27a expression. Also, it inhibited the growth of B16‐based lung metastasis in mice (Xiang et al. 2021). Moreover, delphinidin was able to significantly reduce 12‐O‐tetradecanoylphorbol13‐acetate (TPA)‐induced neoplastic cell transformation in mouse epidermal JB6 *P*+ cells, suggesting its potential role as a skin cancer chemopreventive agent (Kuo et al. 2019). In addition, a study by Singh and Katiyar (2013) revealed that EGCG treatment of A431 and SCC13 human epidermoid carcinoma (squamous cell carcinoma of the skin) cell lines led to reduced cell viability and increased cell death, which were linked to β‐catenin signaling inactivation. Lastly, using human epidermoid carcinoma A431 cells, naringenin was shown to reduce cell viability with concomitant increase in nuclear condensation and DNA fragmentation while increasing ROS production, inducing apoptosis via mitochondrial depolarization and caspase‐3 upregulation, and arresting cell cycle in G_0_/G_1_ phase (Ahamad et al. 2014).

#### 3.1.4 Skin Inflammatory Diseases

Skin is prone to inflammatory processes and diseases, and flavonoids have been reported as potential therapeutic agents for several of them, including psoriasis, atopic dermatitis, and acne vulgaris. In most cases, especially concerning psoriasis and atopic dermatitis, flavonoids exert therapeutic effects mainly through the downregulation of pro‐inflammatory cytokines, resulting in improved tissue features such as reduced erythema, scaling, skin thickness, and lesions. In acne vulgaris' case, most flavonoids' beneficial effects come from the impairment of *Cutibacterium acnes* growth and induced inflammation, leading to similar tissue outcomes like decreased thickness or swelling, but also reduced whiteheads, blackheads, papules, sebum secretion, and porphyrin. These findings reveal flavonoids as potent inhibitors of the most common skin inflammatory conditions.

Regarding psoriasis, in vivo imiquimod‐induced psoriasis animal models have been used to study flavonoids effects in this skin condition. For instance, kaempferol ameliorated psoriasis‐like skin lesions in mice while reducing the populations of dendritic and γδT17 cells and downregulating the expression of pro‐inflammatory cytokines (IL‐23, IL‐17A, TNF‐α, IL‐6, and IL‐1β) and pathways (Janus kinase (JAK)‐signal transducer and activator of transcription (STAT)) (Li, Cui, et al. 2023). Moreover, topical application of pinocembrin significantly improved the skin psoriasis area and severity index score, epidermal thickness, inflammation, hyperplasia, hyperkeratosis, and CD4+ T‐cell infiltration. The pinocembrin‐treated group also reduced the expression of inflammatory cytokines and keratinocyte proliferation markers in dorsal skin. Meanwhile, phosphorylated STAT (pSTAT) 3 levels decreased (Huang et al. 2022). Furthermore, formononetin was reported to improve psoriatic pathological features (erythema, scale, thickness of skin lesions), while reducing interferon (IFN)‐α, IFN‐β, and IFN‐γ expression, as well as TNF‐α and IL‐17 inflammatory factors. It also suppressed the production of IFN‐related chemokines CXCL9, CXCL10, CXCL11, and chemokine (C‐X‐C motif) receptor (CXCR) 3, and transcription factors p‐STAT1, p‐STAT3, and IFN regulatory factor 1 (Xu et al. 2024).

Regarding atopic dermatitis, an in vivo 2,4‐dinitrochlorobenzene‐induced atopic dermatitis BALB/c mouse model has been used to demonstrate that luteolin reduces immunoglobulin E (IgE) serum levels while improving the architecture of injured skin tissue. Also, it reduces oxidative stress and inflammation, as well as the number of white blood cells (Wang et al. 2021). Additionally, myricetin was capable of reducing serum IgE and histamine, inhibited the infiltration of CD 4+ T‐cells, and modulated the expression of cytokines and pro‐inflammatory factors in an in vivo dinitrofluorobenzene‐induced atopic dermatitis KM mouse model. Meanwhile, it restored impaired barrier function via the upregulation of filaggrin's mRNA and protein expression (Gao, Tang, et al. 2023). Finally, another study reported that hesperidin was able to reduce skin lesions and serum IgE levels, as well as the expression of the inflammatory markers IL‐17 and IFN‐γ, in NC/Nga mice, a human‐like mouse model of atopic dermatitis (Nagashio et al. 2013).

Regarding acne vulgaris, in *C. acnes* ‐stimulated HaCaT cells, phloretin effectively inhibited the growth of *C. acnes* and reduced the *C. acnes*‐induced TLR 2‐mediated inflammation. Also, phloretin was capable of binding to *C. acnes* β‐ketoacyl‐acyl carrier protein (ACP) synthase III (KAS III), leading to reduced fatty acid synthesis and consequently impairing bacterial survivability (Cheon et al. 2019). The antibacterial activity of phloretin against *C. acnes* and its protective role over *C. acnes* ‐induced HaCaT cells has also been documented by Kum et al., whose results revealed that phloretin inhibited *C. acnes* growth and prevented bacterial‐induced inflammation in HaCaT cells by reducing COX2 promoter and prostaglandin E_2_ (PGE2) expression. Moreover, in 1 month of placebo‐controlled trials, phloretin reduced comedo counts and sebum output with both clinical and statistical significance. Also, after 4 weeks of treatment, the phloretin‐treated group experienced significant reductions in whiteheads, blackheads, papules, sebum secretion, and porphyrin levels compared to before treatment (Kum et al. 2016). In another study, Lim et al. reported that quercetin ameliorated *C. acnes* ‐induced inflammation in HaCaT, THP‐1, and RAW 264.7 cells. Additionally, using an in vivo *C. acnes* intradermal injection BALB/c mouse model, quercetin was able to reverse some *C. acne*s‐injection effects by markedly reducing ear thickness and swelling (Lim et al. 2021).

#### 3.1.5 Other Activities

Besides the aforementioned effects of flavonoids on the skin, some others are less commonly explored. For instance, pinocembrin has been reported to ameliorate skin fibrosis by inhibiting the proliferation, migration, and invasion of keloid fibroblasts and mouse primary dermal fibroblasts. Furthermore, in vivo experiments demonstrated that pinocembrin effectively alleviated bleomycin‐induced skin fibrosis and reduced the gross weight and fibrosis‐related protein expression in keloid tissues of mice. This study suggested that the therapeutic effects were due to the suppression of TGF‐β1/Smad (small mother against decapentaplegic) signaling and attenuation of TGF‐β1‐induced activation of skin fibroblasts (Li, Zhai, et al. 2021). In another study, using an in vivo hydroquinone‐treated mouse model, apigenin was shown to ameliorate vitiligo, “a chronic autoimmune disease characterized by depigmented white patches on the skin caused by the depletion of melanocytes” (Akl et al. 2024), by reducing depigmentation, inflammatory markers, and oxidative stress, and increasing tyrosinase (important for melanogenesis process). Also, it increased melanin‐containing hair follicles and decreased the expression of non‐phosphorylated p38 mitogen‐activated protein kinases (MAPK) (Chauhan et al. 2024).

### 3.2 Impact of Flavonoids on the Molecular Mechanisms (“Hallmarks”) of Aging

The mechanisms that affect skin aging are yet poorly understood. It is known that aging is dependent on several molecular mechanisms, known as “hallmarks”, which are the core underlying machinery for how our bodies age (López‐Otín et al. 2013; López‐Otín, Blasco, et al. 2023; López‐Otín, Pietrocola, et al. 2023). López‐Otín et al. (2013) listed and described nine tentative hallmarks of aging, representing common denominators of aging in distinct organisms, especially mammals. These hallmarks were genomic instability, telomere attrition, epigenetic alterations, loss of proteostasis, deregulated nutrient sensing, mitochondrial dysfunction, cellular senescence, stem cell exhaustion, and altered intercellular communication. Nonetheless, 10 years later, López‐Otín, Blasco, et al. (2023) updated their notion of these mechanisms and described three new hallmarks: disabled macro‐autophagy, chronic inflammation, and dysbiosis. As shown in Figure 2, altogether, these 12 hallmarks are usually categorized under 3 groups: primary (genomic instability, loss of proteostasis, epigenetic alterations, telomere attrition, disabled macroautophagy), antagonistic (deregulated nutrient‐sensing, mitochondrial dysfunction, cellular senescence), and integrative (stem cell exhaustion, altered intercellular communication, chronic inflammation, dysbiosis) (López‐Otín, Blasco, et al. 2023; Baechle et al. 2023; Biga et al. 2025; Tartiere et al. 2024). Although these mechanisms may seem independent at first glance, it would be a mistake to assume this with certainty. In fact, it is precisely on their interdependence that the classification first proposed by López‐Otín et al. (2013) is founded: *primary* causes of age‐related damage, *antagonistic* responses to damage, and *integrative* phenotypic causes (Costa et al. 2025). As an example, telomere attrition is a fundamental cause of cellular senescence, potentially leading to chronic inflammation (Zhu et al. 2019).

**FIGURE 3:**
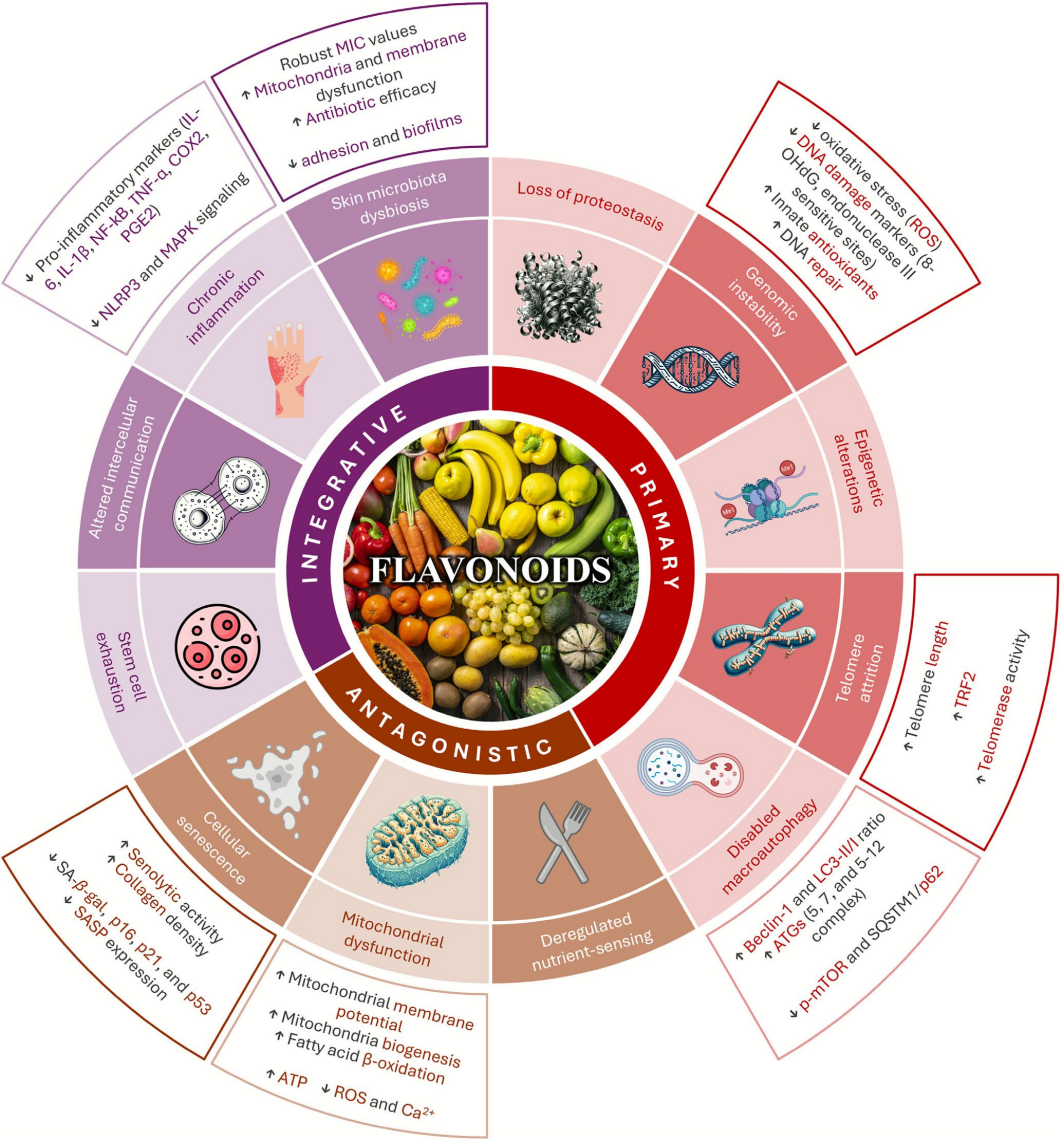
Categorization of the hallmarks of aging and brief description of the flavonoids' impact on them. The schematic representation combines the 12 hallmarks of aging proposed in the literature: genomic instability, telomere attrition, epigenetic alterations, loss of proteostasis, disabled macroautophagy, deregulated nutrient‐sensing, mitochondrial dysfunction, cellular senescence, stem cell exhaustion, altered intercellular communication, chronic inflammation, and (skin microbiota) dysbiosis. These mechanisms are classified into three groups: Primary, antagonistic, and integrative. Additionally, a summary of the effects of flavonoids on several of these hallmarks is provided.

In this section, we aim to describe the impact of flavonoids on cellular aging by examining their effects on specific aging mechanisms, including genomic instability (DNA damage), telomere attrition, mitochondrial dysfunction, cellular senescence, chronic inflammation, disabled macro‐autophagy, and skin microbiota dysbiosis. A summary of these effects is presented in Figure 3 and Table 2.

**TABLE 2:** Effect of flavonoids in the hallmarks of aging.

Flavonoid	Subclass	Model(s)	Effect/impact	References
Genomic instability (DNA damage)				
Apigenin	Flavone	In vitro edifenphos‐induced DNA damage in human peripheral blood lymphocytes	Reduced oxidative stress, DNA damage, and apoptosis	(Ahmad et al. 2019)
Quercetin	Flavonol	In vivo DMH‐induced colon carcinogenesis Wistar rat model	Suppression of DNA damage and induction of DNA repair; increase of enzymatic and nonenzymatic antioxidants	(Darband et al. 2020)
Myricetin	Flavonol	In vivo arsenite‐induced DNA damage BALB/c mouse model (natural killer cells)	Decreased DNA damage by reducing oxidative stress and preserving poly(ADP‐Ribose) polymerase 1 activity	(Ma et al. 2021)
Hesperidin	Flavanone	In vivo streptozotocin‐diabetes mellitus‐induced testicular DNA damage Sprague–Dawley rat model	Decreased DNA damage and oxidative stress levels; increased enzymatic and nonenzymatic antioxidants	(Aksu et al. 2021)
Naringenin	Flavanone	In vivo potassium oxonate‐induced Hyperuricemia Wistar rat model (liver tissue)	Decreased DNA damage (↓ 8‐OHdG)	(Calis et al. 2022)
Epigallocatechin‐3‐gallate	Flavanol	In vitro bleomycin‐induced DNA damage in human leucocytes	Decreased DNA damage (↓ breaks, ↓ endonuclease III sensitive sites)	(Glei and Pool‐Zobel 2006)
Cyanidin	Anthocyanidin	In vitro cisplatin‐induced DNA damage in HK‐2 proximal tubular cells	Decreased DNA damage (↓ DNA strand breaks, ↓ intracellular ROS)	(Gao et al. 2013)
Phlorizin	Chalcone	In vitro H_2_O_2_‐induced DNA damage in HepG2 cells	Decreased DNA damage (↓ DNA migration, ↓ intracellular ROS)	(Wang et al. 2019)
Telomere attrition				
Quercetin	Flavonol	Randomized controlled before‐and‐after study with type 2 diabetic patients (human whole‐blood samples)	Increased telomere length	(Mantadaki et al. 2024)
Epigallocatechin‐3‐gallate	Flavanol	In vivo heart/muscle‐specific deletion of MnSOD‐induced heart failure mouse model	Prevented telomere shortening, and increased telomerase activity	(Oyama et al. 2017)
		In vivo abdominal aortic constriction‐induced cardiac hypertrophy Sprague–Dawley rat model (cardiomyocytes)	Prevented telomere shortening and telomere repeat‐binding factor 2 (TRF2) loss	(Sheng et al. 2013)
Mitochondrial dysfunction				
Pelargonidin	Anthocyanidin	In vivo reserpine‐induced neuronal mitochondrial dysfunction Wistar rat model	Improved neuronal mitochondrial function (↑ complex I activity)	(Rashed et al. 2023)
Pinocembrin	Flavanone	In vitro H_2_O_2_‐induced mitochondrial dysfunction SH‐SY5Y cell model	Prevented mitochondrial depolarization and ATP decrease; attenuated redox impairment in mitochondrial membranes; enhanced tricarboxylic acid (TCA) cycle enzymes	(de Oliveira et al. 2018)
Eriodictyol	Flavanone	In vitro hypoxia/reoxygenation‐induced cardiomyocytes (H9c2) injury cell model	Suppressed the overload of intracellular Ca^2+^; prevented ROS overproduction; blocked mitochondrial permeability transition pore opening, and increased mitochondrial membrane potential; decreased ATP depletion	(Xie et al. 2019)
Formononetin	Isoflavone	In vivo isoproterenol‐induced cardiac fibrosis C57BL/6 mouse model; in vitro isoproterenol‐treated cardiomyocytes	Improved mitochondrial dysfunction by regulating the expressions of ALDH2, HADH, and MAOB; reduced ROS production and preserved mitochondrial membrane potential	(Qian et al. 2024)
Luteolin	Flavone	In vitro H_2_O_2_‐induced mitochondrial dysfunction human umbilical vein endothelial cell model	Suppressed intracellular Ca^2+^ rise and subsequent mitochondrial membrane potential collapse; inhibited cytochrome c release from mitochondria	(Chen et al. 2020)
Kaempferol	Flavonol	In vitro H_2_O_2_‐induced mitochondrial dysfunction in porcine oocytes	Prevented mitochondrial membrane potential collapse; reduced ROS production	(Yao et al. 2019)
(+)‐Catechin	Flavanol	In vitro methylglyoxal‐induced mitochondrial dysfunction EA.hy926 cell model	Prevented mitochondrial membrane potential collapse; inhibited mitochondrial swelling	(Zhang et al. 2017)
Phloretin	Chalcone	In vitro palmitic acid (PA)‐induced HepG2 cell model; In vivo high fat diet‐induced hepatic steatosis C57BL/6J mouse model	Promoted mitochondrial biosynthesis; inhibited mitochondrial swelling; promoted fatty acid β‐oxidation	(Han et al. 2021)
Cellular senescence				
Fisetin	Flavonol	Murine/human cell/tissue types (e.g., MEFs/IMR90 cells; adipose tissue)	Exerted potent senolytic activity: restored tissue homeostasis, reduced age‐related pathology, and extended lifespan	(Yousefzadeh et al. 2018)
Malvidin	Anthocyanidin	In vitro H_2_O_2_‐induced premature senescence in human normal embryonic lung‐derived diploid fibroblasts (WI‐38)	Reduced senescence markers (↓ p53, ↓ p21); increased cellular lifespan	(Seo et al. 2016)
Apigenin	Flavone	In vitro H_2_O_2_/doxorubicin‐induced senescence in WI‐38 fibroblasts	Promoted cell growth; reduced senescence markers (SA‐B‐gal, acetyl (ac)‐p53, p21, and p16)	(Li, Zhu, et al. 2021)
Fisetin	Flavonol	In vitro doxorubicin/long‐term passaging/ionizing radiation‐induced senescence in human dermal fibroblasts (HDFs); in vivo aged skin xenograft mouse model	Eliminated senescent cells in all in vitro models; increased collagen density and decreased senescence‐associated secretory phenotypes (SASP) in vivo (↓ MMPs, ↓ ILs)	(Takaya et al. 2024)
Hesperetin	Flavanone	In vitro D‐galactose‐induced aged SH‐SY5Y cells	Decreased SA‐B‐gal, p16, and p21	(Lee, Kim, et al. 2020)
Genistein	Isoflavone	In vitro ox‐LDL‐induced senescence in HUVECs	Decreased SA‐B‐gal, p16, and p21	(Zhang, Yang, et al. 2019)
(−)‐Epicatechin	Flavanol	High passage (aged) bovine coronary artery endothelial cells	Decreased SA‐B‐gal	(Ramirez‐Sanchez et al. 2018)
Chronic inflammation				
Chrysin	Flavone	In vivo lead acetate‐induced kidney inflammation Sprague–Dawley rat model	Ameliorated inflammation (↓ NF‐κB, ↓ IL‐33, ↓ PGE‐2, ↓ TNF‐α)	(Kucukler et al. 2021)
Isoliquiritigenin	Chalcone	In vitro *Mycobacterium tuberculosis* ‐induced inflammation raw 264.7/murine primary peritoneal macrophages cell models	Inhibited IL‐1β, TNF‐α, and IL‐6 secretion; inhibited gasdermin D activation; suppressed NLRP3 inflammasome	(Sun et al. 2022)
Fisetin	Flavonol	In vivo LPS‐induced septic acute kidney injury C57BL/6 J mouse model	Inhibited renal expression of IL‐6, IL‐1β, TNF‐α, HMGB1, iNOS and COX‐2	(Ren et al. 2020)
Naringin	Flavanone	In vivo acrylamide‐induced nephrotoxicity Sprague–Dawley rat model	Decreased TNF‐α, IL‐1β, IL‐6, NF‐κB, IL‐33, and COX‐2	(Gelen et al. 2022)
Daidzein	Isoflavone	In vitro LPS‐ induced inflammation in primary mouse hepatocytes	Decreased IL‐1β, IL‐6 and TNF‐α	(Yu et al. 2020)
(−)‐Epicatechin	Flavanol	In vivo LPS‐induced renal inflammation Sprague Dawley rat model	Decreased the expression of TNF‐α, iNOS and IL‐6	(Prince et al. 2017)
Delphinidin	Anthocyanidin	In vivo spinal cord injury‐induced inflammation Sprague–Dawley rat model	Inhibited TNF‐α, IL‐6, COX‐2, and PGE‐2	(Wang et al. 2017)
Disabled macro‐autophagy				
Apigenin	Flavone	In vivo chronic restraint‐induced depression BALB/c mouse model	Increased LC3‐II/I and decreased p62 levels; increased the expression of AMPK and Unc‐51 like autophagy activating kinase‐1; inhibited mTOR levels	(Zhang, Bu, et al. 2019)
Kaempferol	Flavonol	In vivo middle cerebral artery occlusion‐reperfusion‐ induced cerebral injury Sprague–Dawley rat model	Increased LC3‐II/I ratio and Beclin1, and decreased p62 levels; increased levels of ATG4, ATG5 and ATG7	(Yuan et al. 2022)
Pinocembrin	Flavanone	In vitro glucocorticoid‐induced apoptosis in osteocyte‐like MLO‐Y4 cells	Increased Beclin1 and LC3‐II/I levels; decreased p62 expression	(Wang et al. 2020)
Genistein	Isoflavone	In vivo streptozotocin‐induced Alzheimer's disease Wistar rat model	Increased LC3‐II/I level; enhanced lysosomal biogenesis and increased levels of TFEB	(Pierzynowska et al. 2019)
Epigallocatechin‐3‐gallate	Flavanol	In vitro H_2_O_2_‐induced oxidative stress in HUVECs	Upregulated the levels of ATG5, ATG7, LC3‐II/I, and the ATG5‐ATG12 complex; knocked down mTOR	(Meng et al. 2020)
Isoliquiritigenin	Chalcone	In vitro hypoxia/re‐oxygenation‐induced myocardial damage in H9c2 cells; in vivo left anterior descending (LAD) coronary artery ligation‐induced ischemia/reperfusion Sprague Dawley rat model	Enhanced Beclin1, LC3II/LC3I, and p‐AMPK/AMPK levels; inhibited p62, and p‐mTOR/mTOR protein expression	(Shen et al. 2024)
Skin microbiota dysbiosis				
Apigenin	Flavone	In vitro *C. albicans* ATCC 90028 monoculture	Induced mitochondrial dysfunction (↑ Ca^2+^ uptake, ↑ ROS) induced apoptosis (DNA fragmentation and caspase activation)	(Lee et al. 2019)
			Exhibited strong antifungal activity (MIC: 5 μg/mL); induced membrane dysfunction (↑ cell shrinkage and ↑ leakage of intracellular content); reduced biofilm formation	(Lee et al. 2018)
Quercetin	Flavonol	In vitro *C. acnes* KCTC3314 monoculture	Exhibited strong antibacterial activity (MIC: 31.25 μg/mL)	(Joo et al. 2022)
		In vitro *S. epidermidis* ATCC 35984 monoculture	Reduced cell surface hydrophobicity; inhibited *S. epidermidis* cells adhesion to glass slides; downregulated the intercellular adhesion (ica) locus; reduced the production of polysaccharide intercellular adhesin (PIA)	(Mu et al. 2021)
Hesperetin laurate	Flavanone derivate	In vitro *Malassezia furfur* KCTC 7545 monoculture	Exerted antifungal activity (MIC: 1 mg/mL)	(Lee et al. 2024)
(+)‐Catechin	Flavanol	In vitro *M. furfur* monoculture	Exerted antifungal activity (MIC: 16–64 μg/mL)	(Aoshima et al. 2009)
Phloretin	Chalcone	In vitro *S. epidermidis* ATCC 12228 monoculture	Exhibited antibacterial activity (MIC: 0.25 mg/mL)	(Kum et al. 2016)
Isoliquiritigenin	Chalcone	In vitro MRSA monocultures; in vivo MRSA‐infected Swiss mouse model	Inhibited bacterial growth; exhibited antibacterial activity (MIC: 50–100 μg/mL); reduced the MIC of β‐lactam antibiotics up to 16‐fold; enhanced oxacillin efficacy in lowering the microbial burden in blood, liver, kidney, lung and spleen tissues	(Gaur et al. 2016)

#### 3.2.1 Genomic Instability or DNA Damage

The generation of damaging substances is one of the first steps in the biological aging process. The most common type of cell damage is mediated by the production of ROS, a mechanism all respiratory organisms share (Polsky et al. 2022; Aschbacher et al. 2013). In this line, genomic instability could be characterized as a wide range of genetic lesions driven by extrinsic (exogenous chemical, physical, and biological agents) or intrinsic (DNA replication errors, chromosome segregation abnormalities, oxidative processes, and spontaneous hydrolytic reactions) and includes point mutations, deletions, translocations, telomere shortening, single‐ and double‐strand breaks, chromosomal rearrangements, problems in nuclear architecture, and gene disruption caused by the integration of viruses or transposons (López‐Otín et al. 2013; López‐Otín, Blasco, et al. 2023).

Flavonoids, like apigenin, quercetin, myricetin, hesperidin, naringenin, EGCG, cyanidin, and phlorizin, exhibit protector effects against DNA damage, both in vitro and in vivo, via restoring DNA repair capacity, reducing oxidative stress, either by ROS neutralization or innate antioxidants enhancement, and attenuating specific DNA damage markers such as 8‐OHdG, endonuclease III sensitive sites, and strand breaks. These outcomes suggest that flavonoids have potential therapeutic applications in combating DNA damage.

Apigenin has been reported to attenuate oxidative stress, DNA damage, and apoptosis induced by edifenphos in human peripheral blood lymphocytes (Ahmad et al. 2019). Furthermore, using a bleomycin‐induced DNA damage model of human peripheral leukocytes, EGCG was found to attenuate the damage by reducing DNA breaks and endonuclease III‐sensitive sites (Glei and Pool‐Zobel 2006). Moreover, cyanidin attenuated cisplatin‐induced apoptosis on HK‐2 proximal tubular cells by reducing DNA damage while ameliorating ROS generation and DNA strand breaks (Gao et al. 2013). In another study, phlorizin was reported to inhibit the H_2_O_2_‐induced DNA damage on HepG2 cells via reducing apoptosis and ROS production, as well as preventing DNA migration (Wang et al. 2019).

The effect of flavonoids on genomic instability has also been explored using in vivo models. For example, using an in vivo 1,2‐dimethylhydrazine (DMH)‐induced colorectal cancer rat model, quercetin was capable of significantly suppressing DNA damage (↓ 8‐OHdG) and promoting DNA repair, while enhancing non‐enzymatic antioxidant activity and expression levels, and consequently inhibiting ROS production (Darband et al. 2020). In another study, myricetin was shown to protect the natural killer cells of mice exposed to arsenite, thereby alleviating the decrease in cell population and reducing DNA damage. The flavonoid was capable of reducing oxidative stress while preserving the poly(ADP‐ribose) polymerase 1 (PARP‐1) activity (Ma et al. 2021). Moreover, in an in vivo streptozotocin‐induced diabetes mellitus rat model with testicular DNA damage, hesperidin allowed to decrease DNA damage levels (↓ 8‐OHdG) while reducing ROS production and enhancing the non‐enzymatic antioxidant activity and expression levels (Aksu et al. 2021). Lastly, naringenin has been reported to decrease DNA damage (↓ 8‐OHdG) while decreasing caspase‐3 and inducing GPX levels in the liver tissue of potassium oxonate‐induced rats (Calis et al. 2022).

#### 3.2.2 Telomere Attrition

DNA damage at the ends of chromosomes (telomeres) is linked to aging and age‐related diseases (Blackburn et al. 2015). Replicative DNA polymerases cannot finish the copy of eukaryotic DNA telomere regions. As a result, after multiple rounds of cell division, telomeres shorten dramatically, leading to genomic instability and, ultimately, apoptosis or cell senescence. Telomerase, an active ribonucleoprotein that elongates telomeres to maintain their appropriate length, has a reverse‐transcriptase activity that can prevent these deleterious repercussions (Blasco 2005; Chakravarti et al. 2021). Nonetheless, telomere attrition is a normal part of human aging (López‐Otín et al. 2013; López‐Otín, Blasco, et al. 2023; Blasco 2007).

In terms of how flavonoids affect it, this mechanism may be the least studied. Nonetheless, some research has demonstrated that quercetin and EGCG inhibit telomere attrition through a variety of mechanisms, such as the preservation of telomere repeat‐binding factor 2 (TRF_2_) or improved telomerase activity. These brief findings highlight the potential telomere‐protective role of flavonoids.

In a randomized controlled before‐and‐after study with type 2 diabetic patients, quercetin intake significantly increased the mean telomere length of human whole‐blood samples (Mantadaki et al. 2024). Furthermore, in an in vivo study where heart failure was induced by heart/muscle‐specific deletion of MnSOD (manganese superoxide dismutase) in mice, Oyama et al. (2017) reported that EGCG was able to prevent the shortening of telomeres and the decrease of telomerase activity. Moreover, telomere attrition was induced in mice using an in vivo abdominal aortic constriction‐induced cardiac hypertrophy model, which was prevented by EGCG, along with the preservation of TRF_2_ (Sheng et al. 2013).

#### 3.2.3 Mitochondrial Dysfunction

Mitochondrial function deteriorates with age due to several linked factors, including the accumulation of mtDNA mutations and impaired proteostasis, which leads to the instability of respiratory chain complexes, decreased organelle turnover, and alterations in mitochondrial dynamics, thereby increasing electron leakage and reducing ATP synthesis. This state disrupts mitochondrial bioenergetics, generates ROS, and can cause membrane permeability, leading to inflammation and cell death (López‐Otín et al. 2013; López‐Otín, Blasco, et al. 2023; Amorim et al. 2022; Green et al. 2011). Logically, mitochondrial activity is essential for maintaining health, and its steady decline contributes to the aging phenotype (López‐Otín, Blasco, et al. 2023).

Flavonoids show significant potential in protecting mitochondria health against external aggressors. In vitro research has shown that these compounds protect mitochondria through several mechanisms like ROS attenuation, enzyme activation, preservation of mitochondria membrane potential, and prevention of Ca^2+^ overflow, concomitant with increased ATP and mitochondria biogenesis, and decreased swelling. In vivo studies further support this as flavonoids, like pelargonidin, formononetin, and phloretin, promote mitochondrial health by modulating cellular mechanisms such as complex I activation, aldehyde dehydrogenase 2 (ALDH2), 3‐hydroxyacyl‐CoA dehydrogenase (HADH), and monoamine oxidase B (MAOB) regulation, and augmented fatty acid β‐oxidation. These results feature flavonoids as strong mitochondria protective phytochemicals.

Concerning in vitro research, pinocembrin has been reported to alleviate mitochondrial dysfunction induced by H_2_O_2_ in SH‐SY5Y cells by preventing mitochondrial depolarization and ATP decrease, attenuating redox impairment in mitochondrial membranes, and enhancing tricarboxylic acid (TCA) cycle enzymes, such as aconitase, α‐ketoglutarate dehydrogenase, and succinate dehydrogenase (de Oliveira et al. 2018). In another study, eriodictyol improved mitochondrial health, thereby preventing hypoxia/reoxygenation‐induced damage in H9c2 cardiomyocytes. This study revealed that the tested flavonoid suppressed the overload of intracellular Ca^2+^, prevented ROS overproduction, blocked mitochondrial permeability transition pore opening, increased mitochondrial membrane potential, and decreased ATP depletion (Xie et al. 2019). Additionally, H_2_O_2_ effects on HUVECs, like intracellular Ca^2+^ rise and subsequent mitochondrial membrane potential collapse and cytochrome c release from mitochondria, were markedly prevented by pretreatment with luteolin (Chen et al. 2020). Moreover, kaempferol was also able to reverse the negative effects of H_2_O_2_ on mitochondria by preventing mitochondrial membrane potential collapse and reducing ROS production in porcine oocytes (Yao et al. 2019). Furthermore, (+)‐catechin was reported to alleviate methylglyoxal‐induced mitochondrial dysfunction in EA.hy926 cells by preventing mitochondrial membrane potential collapse and inhibiting mitochondrial swelling (Zhang et al. 2017). In addition, using two models: (1) in vitro palmitic acid (PA)‐induced HepG2 cell model and (2) in vivo high fat diet‐induced hepatic steatosis C57BL/6J mouse model, phloretin was shown to ameliorate hepatic steatosis by promoting fatty acid β‐oxidation, which was closely related to its ability to improve mitochondrial health through the promotion of mitochondrial biosynthesis and inhibition of mitochondrial swelling (Han et al. 2021).

Other in vivo studies have also described the impact of flavonoids on mitochondria. For instance, using an in vivo reserpine‐induced neuronal mitochondrial dysfunction Wistar rat model, pelargonidin ameliorated mitochondrial function by elevating the activity of complex 1, which is associated with a low ADP/ATP ratio (Rashed et al. 2023). Moreover, formononetin was hypothesized as being able to improve mitochondrial dysfunction in mice/rats with isoproterenol‐induced cardiac fibrosis by regulating the expressions of ALDH2 (↑), HADH (↑), and MAOB (↓) while reducing ROS production and preserving mitochondrial membrane potential in cardiomyocytes (Qian et al. 2024).

#### 3.2.4 Cellular Senescence

Cellular senescence is a state of permanent cell growth/cycle arrest caused by acute or chronic damage (Gorgoulis et al. 2019). It is characterized by stereotyped phenotypic changes and inability to proliferate (López‐Otín et al. 2013; López‐Otín, Blasco, et al. 2023; Campisi and d'Adda di Fagagna 2007; Collado et al. 2007; Hayflick and Moorhead 1961; Kuilman et al. 2010). Senescent cells grow at varying rates in people, ranging from 2 to 20‐fold when comparing young (35 years) and old (> 65 years) healthy donors (Tuttle et al. 2020), mainly affecting fibroblasts, endothelial cells, and immune cells. Nonetheless, all cell types can experience senescence as they age (Xu et al. 2022), which is at least partially caused by telomere shortening with age (Blasco 2005). The most compelling evidence for cellular senescence's role in aging is that sustained eradication of senescent cells extended the health span and lifespan of normally aged mice (Xu et al. 2018).

Flavonoids, like fisetin, malvidin, apigenin, hesperetin, genistein and (−)‐epicatechin, exhibit strong anti‐senescence effects either by reducing specific molecular markers such as SA‐β‐gal, p21, p16, and p53, or by exerting senolytic activity, thus selectively eliminating senescent cells in vitro. Also, fisetin was shown to attenuate the senescence‐associated secretory phenotype (SASP) in vivo, further supporting the previous results. These outcomes reveal flavonoids as potential therapeutic agents against cellular senescence.

Fisetin was shown to exert potent senolytic activity in multiple tissues and cell types, leading to restored tissue homeostasis, reduced age‐related pathology, and extended lifespan (Yousefzadeh et al. 2018). Using an in vitro H_2_O_2_‐induced premature senescence human normal embryonic lung‐derived diploid fibroblasts (WI‐38) cell model, malvidin was able to extend the cellular lifespan while reducing senescence markers such as p53 and p21 (Seo et al. 2016). Furthermore, using a similar model but also testing doxorubicin as an oxidative stress (senescence) inducer on WI‐38 cells, apigenin was shown to reduce SA‐β‐gal activity and promote cell proliferation while decreasing the levels of several senescence markers like acetyl (ac)‐p53, p21, and p16 (Li, Zhu, et al. 2021). Additionally, in a study where senescence was induced in neuronal cells (SH‐SY5Y) by exposure to D‐galactose in vitro, hesperetin was found to ameliorate the cellular aging phenotype by reducing the number of SA‐β‐gal stained cells, as well as downregulating the expression of p16 and p21 (Lee, Kim, et al. 2020). Moreover, genistein was reported to ameliorate senescence on ox‐LDL‐treated HUVECs, reducing the activity of SA‐β‐gal and the expression levels of p16 and p21 proteins (Zhang, Yang, et al. 2019). In a slightly different study, since the senescence model relied on naturally aged (high passage) cells, that is, replicative senescence, (−)‐epicatechin had the capacity to alleviate the senescent state of bovine coronary artery endothelial cells, with decreased activity of SA‐β‐gal (Ramirez‐Sanchez et al. 2018). In another study, senescence was induced in human dermal fibroblasts by either high passaging (replicative senescence), ionizing radiation exposure, or doxorubicin treatment. In all three cases, fisetin could selectively eliminate senescent fibroblasts while attenuating the SASP by decreasing IL‐1α, IL‐6, MMP‐3, and MMP‐9 levels. In addition, fisetin also reduced the amount of SA‐β‐gal positive cells and the expression of senescence‐associated proteins p16 and p53. Furthermore, the authors also used an in vivo aged human skin xenograft mouse model. The results revealed that fisetin was once again capable of reducing senescent cells, concomitant with fewer SA‐β‐gal activity and p16 expression, while reducing SASP (IL‐1α, IL‐6, TNF‐α, MMP‐3, MMP‐9), and improving aged skin phenotype by increasing collagen density and alleviating inflammatory cell infiltrates in the dermis (Takaya et al. 2024).

#### 3.2.5 Chronic Inflammation

Inflammation worsens with age (“inflammaging”), causing systemic symptoms and abnormal local phenotypes. As people age, their circulation levels of inflammatory cytokines and biomarkers (such as C‐reactive protein—CRP) increase (López‐Otín et al. 2013; López‐Otín, Blasco, et al. 2023; Franceschi and Campisi 2014). For example, higher IL‐6 levels in plasma are an accurate biomarker of all‐cause mortality in aging human populations (Hirata et al. 2020). Furthermore, higher inflammation is associated with a decrease in immunological function (Mogilenko et al. 2021). Importantly, inflammation is a major cause of cellular damage, which is thought to accelerate aging (Franceschi and Campisi 2014).

Flavonoids exhibit significant anti‐inflammatory activity by modulating key inflammatory pathways. In vitro studies have shown that compounds such as isoliquiritigenin and daidzein reduce pro‐inflammatory cytokines and suppress NF‐kB (nuclear factor‐kappa B), MAPK, and NLRP3 (NOD‐, LRR‐ and pyrin domain‐containing protein 3) signaling. In vivo, research further confirms these effects, with chrysin, fisetin, naringin, and (−)‐epicatechin alleviating kidney inflammation by inhibiting the expression of inflammatory markers, while delphinidin exhibits similar outcomes for spinal cord inflammation. These findings highlight the potential of flavonoids as therapeutic agents for chronic inflammation.

Isoliquiritigenin was able to attenuate the *Mycobacterium tuberculosis* ‐induced inflammation on Raw 264.7 and murine primary peritoneal macrophage cells by inhibiting the Notch1/NF‐κB and MAPK signaling pathways, concomitant with decreased gasdermin D and IL‐1β levels via suppression of NLRP3 inflammasome, as well as decreased IL‐6, TNF‐α, inducible nitric oxide synthase (iNOS), and COX2 expression (Sun et al. 2022). Moreover, using an in vitro LPS‐induced inflammation primary mouse hepatocyte cell model, daidzein was shown to alleviate inflammation by reducing the expression of inflammatory factors such as IL‐1β, IL‐6, and TNF‐α (Yu et al. 2020).

Regarding in vivo research, chrysin had the capacity to ameliorate kidney inflammation induced by lead acetate in rats, allowing a decrease in the levels of several inflammatory markers, which included NF‐κB, IL‐33, PGE2, and TNF‐α, while reducing COX2 and iNOS activities (Kucukler et al. 2021). Additionally, and also focusing on kidney inflammation, Ren et al. (2020) reported that fisetin was able to alleviate LPS‐induced inflammatory symptoms in mice by inhibiting the renal expression of IL‐6, IL‐1β, TNF‐α, High Mobility Group Box 1 (HMGB1), iNOS, and COX2, thus improving the inflammatory response. Sticking with kidney research and using an in vivo acrylamide‐induced nephrotoxicity Sprague–Dawley rat model, naringin was reported to allow the reduction of inflammatory markers such as TNF‐α, IL‐1β, IL‐6, NF‐κB, IL‐33, and COX2, in kidney tissue (Gelen et al. 2022). Furthermore, (−)‐epicatechin also reduced kidney inflammation induced by LPS treatment in rats by decreasing the expression of TNF‐α, iNOS, and IL‐6 (Prince et al. 2017). Lastly, using an in vivo spinal cord injury‐induced inflammation Sprague–Dawley rat model, delphinidin was able to counteract the adverse effects by reducing the levels of TNF‐α, IL‐6, NF‐κB, COX2, and PGE2 (Wang et al. 2017).

#### 3.2.6 Disabled Macro‐Autophagy

Macro‐autophagy (abbreviated “autophagy”) is the sequestration of cytoplasmic material in two‐membrane vesicles known as autophagosomes, which eventually fuse with lysosomes to destroy luminal substance (Levine and Kroemer 2019). Autophagy declines with age and is one of the most fundamental processes of decreasing organelle turnover, prompting its recognition as a new aging marker. Nonetheless, autophagy genes and proteins are also involved in additional degradation processes, such as LC3 (microtubule‐associated protein light chain 3)‐associated phagocytosis of extracellular material (Galluzzi and Green 2019), and the ejection of intracellular trash (e.g., defective mitochondria) in the form of exospheres for subsequent clearance by macrophages (Nicolás‐Ávila et al. 2020). Even so, strong evidence has been found that autophagy plays a crucial role in aging (López‐Otín, Blasco, et al. 2023).

Flavonoids, like apigenin, kaempferol, pinocembrin, genistein, EGCG, and isoliquiritigenin, have shown capacity to prevent autophagy impairment mainly by activating relevant markers such as LC3‐II/I ratio, beclin‐1, and autophagy‐related proteins (ATGs), and decreasing p62 and mTOR (mammalian target of rapamycin) expression, while enhancing AMPK (AMP‐activated protein kinase) signaling, both in vitro and in vivo. According to these findings, flavonoids can be considered as potential autophagy activators.

Pinocembrin was able to increase Beclin‐1 and LC3B‐II levels, and decrease p62 expression, thus indicating autophagy activation in an in vitro glucocorticoid‐induced apoptosis osteocyte‐like MLO‐Y4 cell model (Wang et al. 2020). Furthermore, EGCG was found to upregulate the levels of ATG5, ATG7, LC3‐II/I, and the ATG5‐ATG12 complex while knocking down mTOR, thereby inducing autophagy after H_2_O_2_ treatment in HUVECs (Meng et al. 2020). In another study, the effect of isoliquiritigenin on the autophagy process in vitro in H9c2 cells and in vivo in Sprague–Dawley rats, following hypoxia/re‐oxygenation‐induced and left anterior descending coronary artery ligation‐induced myocardial injury, respectively, was explored. The study's results revealed that the tested flavonoid was capable of inducing autophagy by enhancing Beclin‐1, LC3‐II/I, and p‐AMPK/AMPK levels, concomitant with the inhibition of p62 and p‐mTOR/mTOR protein expression, in both used models (Shen et al. 2024). Moreover, using an in vivo chronic restraint‐induced depression BALB/c mouse model, apigenin was shown to allow the induction of autophagy in the hippocampus by increasing the levels of LC3‐II/I and the expression of AMPK and Unc‐51 like autophagy activating kinase‐1, while decreasing p62 levels, and inhibiting mTOR expression (Zhang, Bu, et al. 2019). Furthermore, genistein was reported to ameliorate behavioral and biochemical defects in a streptozotocin‐induced sporadic Alzheimer's disease (AD) rat model, and the authors suggest that the observed therapeutic effects were autophagy‐dependent as genistein was able to increase LC3‐II/I level and to enhance lysosomal biogenesis, which was corroborated by the increased levels of transcription factor EB (TFEB) while reducing the levels of AD‐related pathogenic proteins APP (amyloid precursor protein) and βA (beta‐amyloid) (Pierzynowska et al. 2019). In addition, kaempferol was able to activate autophagy in a cerebral ischemia/reperfusion (I/R)‐induced injury rat model, as confirmed by an increased LC3‐II/I ratio, Beclin‐1, ATG4, ATG5 and ATG7 levels, and decreased p62 expression (Yuan et al. 2022).

#### 3.2.7 Skin Microbiota Dysbiosis

Skin microbiota is predicted to play an essential part in and be affected by the hallmarks of aging since it is responsible for the host skin's homeostasis and protection (Baldwin et al. 2017; Chen et al. 2022). However, López‐Otín, Blasco, et al. (2023) exclusively examine gut microbiota, ignoring the potential impact of skin microbial flora on aging. Research has linked changes in skin microbiota composition to aging skin (Abadías‐Granado et al. 2021; Boxberger et al. 2021; Boyajian et al. 2021; Ratanapokasatit et al. 2022). However, there is a lack of studies linking these findings to skin aging mechanisms, which requires further investigation. Besides that, skin microbiota changes are influenced by a variety of factors other than age, including skin site, ethnicity, gender, skin illnesses, lifestyle (UV exposure, smoking), and pollution (Lee, Watson, and Kleyn 2020; Boxberger et al. 2021; Wu et al. 2020). To these factors, we can add the use of cosmetics, whose active ingredients, such as flavonoids, may have an impact on skin microbiota balance. In this line of thought, it is important to understand the kind of effect these polyphenolic compounds may exert over skin‐related microorganisms.

Flavonoids have been shown to exhibit strong antimicrobial properties against skin‐related bacteria and fungi through mechanisms like oxidative stress, mitochondrial dysfunction, membrane disruption, and biofilm inhibition. Quercetin and phloretin target *Staphylococcus epidermidis* , while isoliquiritigenin exhibits strong inhibition over methicillin‐resistant *Staphylococcus aureus* (MRSA) in vitro and in vivo. Quercetin also exerts inhibitory effects over *C. acnes* , while apigenin, hesperetin laurate, and (+)‐catechin show broad antifungal effects against *Candida albicans* and *Malassezia furfur* strains.

Beginning with *Staphylococcus epidermidis* and using the ATCC35984 strain, quercetin reduced cell surface hydrophobicity, which supported the flavonoid's anti‐biofilm effect, as well as inhibited cell adhesion to glass slides, downregulated the intercellular adhesion (ica) locus and reduced the production of polysaccharide intercellular adhesin (PIA) (Mu et al. 2021). Also, phloretin has been reported to exert antimicrobial activity over *S. epidermidis* ATCC12228 and to inhibit its growth, with a minimum inhibitory concentration (MIC) of 0.25 mg mL^−1^ (Kum et al. 2016). Regarding *C. acnes* , the same study also revealed a MIC of 0.5 mg mL^−1^ over the ATCC11827 strain by the flavonoid phloretin, which was capable of attenuating *C. acnes*‐induced production of COX2 promoter and PGE2 (inflammation) on HaCaT cells. Moreover, quercetin was shown to inhibit *C. acnes* KCTC3314 proliferation, presenting a MIC of 31.25 μg mL^−1^ (Joo et al. 2022). Concerning the *Malassezia* fungal genus, both (+)‐catechin and hesperetin laurate (a hesperetin derivative) showed antifungal activity over *M. furfur* (over several strains in the first case and over KCTC7545 strain in the latter), with MIC values of 16–64 μg mL^−1^ and 1 mg mL^−1^, respectively (Aoshima et al. 2009; Lee et al. 2024). Furthermore, also *Candida albicans* has been reported to be affected by flavonoid (apigenin) treatment. For instance, apigenin had the capacity to induce mitochondrial dysfunction (↑ increased Ca^2+^ uptake and ROS) in the ATCC90028 strain, thereby triggering cellular apoptosis, as observed through DNA fragmentation and caspase activation (Lee et al. 2019). Apigenin could also impact the same *C. albicans* strain by inducing membrane dysfunction (↑ cell shrinkage and ↑ leakage of intracellular content), reducing biofilm formation, and exerting strong antifungal activity, presenting a MIC value of 5 μg mL^−1^ (Lee et al. 2018). About *Staphylococcus aureus* , isoliquiritigenin inhibited bacterial growth over methicillin‐resistant *Staphylococcus* *aureus* (MRSA) and exhibited antibacterial activity with MIC values ranging from 50 to 100 μg mL^−1^. Moreover, it was able to enhance the efficacy of β‐lactam antibiotics by reducing the MIC values up to 16‐fold. In this study, the authors further tested the efficacy of isoliquiritigenin in vivo on MRSA‐infected mice, and the results revealed that oxacillin efficacy was enhanced by flavonoid treatment, as evidenced by a reduction in microbial burden in blood, liver, kidney, lung, and spleen tissues (Gaur et al. 2016).

Having said that, can we be sure that flavonoids slow down the skin aging process? The answer is “we don't know” if we only consider the distinct viewpoint of the molecular mechanisms underlying (skin) cellular aging, since most study cases do not explore skin cells and/or tissue. In fact, only one skin‐related study has been presented (Takaya et al. 2024), which does not imply that no additional skin‐related works have been previously conducted, but it is a strong indicator of the scarcity of research in this field. Nevertheless, some flavonoids have been shown to impact different cells and tissues, which could hold the promise of a potential sustained transfer of these benefits towards the skin. Additionally, the majority of the phenomena described in Section 3.1 have a tight connection to skin aging. For example, it has been estimated that up to 80% of the visible signs of skin aging are caused by UV radiation (Zargaran et al. 2022). Changes in the characteristics of aging skin have also been connected to a higher risk of developing skin cancer, according to researchers at the Johns Hopkins Kimmel Cancer Center (Weeraratna 2024). From our point of view, these findings make it clear that flavonoids may be a promising new approach to attenuate the early signs of skin aging. However, can these positive effects be extended to the mitigation of psychological stress effects, thus helping flavonoids to take the next step on becoming a real alternative to stress‐related skin aging? In the next section, the authors seek to explore the relationship between these phytochemicals and the major psychological stress‐related hormones and mediators.

### 3.3 Interplay Between Flavonoids and Stress Mediators

Given that conventional treatments for mental disorders—including psychological stress and related conditions—have been associated with serious adverse effects such as organ damage, miscarriage, drowsiness, and sexual dysfunction, and their effectiveness has declined due to practical limitation and restricted treatment accessibility (Noor 2024), there is an urgent need to develop novel therapeutic strategies. Consequently, targeted interventions to improve psychological health are critically required (Currie et al. 2023). Flavonoids have emerged as promising candidates for the formulation of new phytochemical‐based drugs and therapies, owing to their extensive range of beneficial biological properties and potential health‐promoting effects. Preclinical studies, mostly, have demonstrated that flavonoids can produce multiple positive outcomes, including improvements in mood, depression, anxiety, cognitive function, and overall psychological well‐being (Currie et al. 2023; Alizadeh et al. 2024; Jia et al. 2023; Pannu et al. 2021). For instance, resveratrol has been shown to alleviate depressive‐like behaviors induced by both physical and psychological stress in a mouse model (Ardianto et al. 2021). In addition, Colombage et al. (2024) found that a two‐week flavonoid‐enriched diet led to greater positive affect and reduced postpartum depression among women in the intervention group.

Accordingly, this section examines whether and how flavonoids influence psychological stress by analyzing their capacity to modulate its primary mediators. This exploration aims to determine whether these phytochemicals genuinely possess therapeutic potential for managing stress‐related psychological disorders, including those linked with aging. The interactions between flavonoids and key stress mediators—such as glucocorticoids (e.g., cortisol), catecholamines, and substance P—will be discussed, with the summarized findings presented in Table 3.

**TABLE 3:** The interplay between flavonoids and stress mediators.

Flavonoid	Subclass	Model(s)	Interplay	References
*Cortisol and glucocorticoids*				
Apigenin Daidzein Genistein Formononetin	Flavone Isoflavone Isoflavone Isoflavone	In vitro di‐buthylyl cAMP‐stimulated H295R cell model	Significantly reduced the production of **cortisol**	(Ohno et al. 2002)
Myricetin	Flavonol	In vivo dexamethasone‐induced osteoporosis Sprague–Dawley rat model	Ameliorated **dexamethasone** effects and alleviated osteoporosis	(Fan et al. 2018)
Butein	Chalcone	In vitro corticosterone‐treated Neuro2A cells	Prevented **corticosterone**‐induced cytotoxicity	(Ohmoto et al. 2020)
Formononetin	Isoflavone	In vivo chronic corticosterone‐induced depression mouse model	Reduced **corticosterone** serum levels; protected against **corticosterone**‐induced effects	(Zhang et al. 2022)
Naringenin Fisetin	Flavanone Flavonol	In vitro 11 beta‐hydroxysteroid dehydrogenase (HSD‐11β)‐inhibition assay	Inhibited **HSD‐11β** (inactivates cortisol to cortisone) with IC_50_ values of 0.34 (naringenin) and 0.47 mM (fisetin)	(Zhang and Wang 1997)
Quercetin	Flavonol	In vivo oxytetracycline‐treated silver catfish	Prevented increased serum levels of **cortisol**	(da Silva Pês et al. 2020)
Kaempferol	Flavonol	In vivo immobilization/swimming‐induced stress Swiss rat models	Restored **cortisol** levels (↓) compared to the stress control group	(Habbu et al. 2010)
Genistein Daidzein	Isoflavones	In vitro ACTH‐stimulated porcine adrenocortical cells	Significantly decreased basal and ACTH‐stimulated **cortisol** production	(Kaminska et al. 2014)
				(Kaminska et al. 2013)
(−)‐Epicatechin	Flavanol	In vitro H_2_O_2_‐induced oxidative stress in human monocyte cell line U937	Reduced **cortisol** resistance, that is, preserved its anti‐inflammatory activity	(Ruijters et al. 2014)
*Catecholamines*				
(−)‐Epicatechin	Flavanol	In vivo isoproterenol‐induced myocardial infarction Wistar rat model	Prevented **isoproterenol**‐induced tachycardia, cardiac hypertrophy, and nuclear factor‐κB inflammatory signaling pathway	(Ponnian 2022)
Malvidin	Anthocyanidin	In vivo isoproterenol‐induced myocardial infarction Wistar rat model	Prevented negative **isoproterenol** effects	(Wei et al. 2017)
Daidzein	Isoflavone	In vivo MPTP‐induced Parkinson's disease C57BL6 mouse model	Increased **dopamine** production levels	(Wu et al. 2022)
Luteolin	Flavone	In vivo spontaneously hypertensive rats	Decreased plasma levels of **norepinephrine** and **epinephrine**	(Gao, Yu, et al. 2023)
Quercetin Fisetin	Flavonols	In vivo epinephrine‐induced lipolysis in rat adipocytes	Potentiated **epinephrine**‐induced lipolysis	(Kuppusamy and Das 1994)
Hesperetin Butein	Flavanone Chalcone		Inhibited **epinephrine**‐induced lipolysis	(Kuppusamy and Das 1992)
Pinocembrin	Flavanone	In vivo collagen/epinephrine‐induced pulmonary thromboembolism mouse model	Inhibited collagen/**epinephrine**‐induced pulmonary thromboembolism	(Li, Liu, et al. 2024)
(−)‐Epicatechin	Flavanol	In vitro epinephrine‐induced platelet aggregation	Decreased the maximal platelet aggregation induced by **epinephrine**	(Sinegre et al. 2019)
Epigallocatechin‐3‐gallate	Flavanol	Randomized, placebo‐controlled, single‐blind, cross‐over trial of graded cycling to volitional exhaustion	Decreased **epinephrine** and **norepinephrine** plasma levels, measured at different stages of the trial	(Churm et al. 2023)
Apigenin	Flavone	In vivo spontaneously hypertensive rats	Attenuated hypertension (↓ plasma **norepinephrine**)	(Gao et al. 2021)
Genistein	Isoflavone	In vivo two‐kidney, one‐clipped (2K1C) hypertensive Sprague–Dawley rats	Alleviated **norepinephrine** plasma levels, concomitant with an array of kidney‐protective effects	(Poasakate et al. 2022)
Myricetin	Flavonol	In vivo single prolonged stress‐induced PTSD‐like symptoms in Sprague–Dawley rats	Restored **norepinephrine** levels (↓) in the fear circuit regions, medial prefrontal cortex, hippocampus and amygdala.	(Sur and Lee 2022)
*Substance P*				
Pinocembrin	Flavanone	In vivo mechanically‐induced hip fracture pain Lewis rat model	Downregulated **Substance P** levels in the ipsilateral L5 dorsal horn segment	(Xing et al. 2022)
Fisetin	Flavonol	In vivo Substance P‐induced chronic urticaria BALB/c mouse model	Prevented urticaria‐like symptoms induced by **Substance P**	(Zhang et al. 2023)
Apigenin	Flavone	In vivo high fat diet‐induced obesity C57BL/6J mouse model	Reduced the expression of **Substance P** in myenteric ganglia of HFD mice	(Gentile et al. 2018)
Quercetin	Flavonol	In vivo loperamide‐induced constipation Sprague–Dawley rat model	Increased serum levels of **Substance P**, thus accelerating intestinal peristaltism and gastric discharge	(Liu and Zhi 2021)
		In vivo berberine‐induced constipation C57BL/6J mouse model		(Cui et al. 2024)
Naringenin	Flavanone	In vivo loperamide‐induced constipation ICR mouse model		(Yin et al. 2018)

#### 3.3.1 Cortisol and Glucocorticoids

Flavonoids exhibit several effects over glucocorticoids' biological impact in several cell types/tissues through various mechanisms such as restoring their normal levels, modulating their production and resistance, or even by directly counteracting their action, thus alleviating their effects.

Myricetin was found to inhibit dexamethasone‐induced osteoporosis in rats by increasing bone mineral density and enhancing osteocalcin (OCN), bone morphogenetic protein 2 (BMP2), runt‐related transcription factor 2 (Runx2), and alkaline phosphatase (ALP). In vitro, results were similar, with myricetin promoting osteoblast differentiation and mineralization in dexamethasone‐treated MC3T3‐E1 cells, concomitant with increases in BMP2, Runx2, ALP, OCN, collagen type I alpha 1, and osteopontin (OPN) levels (Fan et al. 2018). Moreover, butein significantly decreased the levels of corticosterone‐induced ROS in Neuro2A cells, thus preventing cytotoxicity in vitro (Ohmoto et al. 2020). Furthermore, using an in vivo corticosterone‐induced depression mouse model, formononetin was able to reduce serum levels of corticosterone while upregulating the levels of glucocorticoid receptor and brain‐derived neurotrophic factor (BDNF) in the hippocampus. Also, it inhibited corticosterone‐induced neuronal impairment and promoted neurogenesis in the hippocampus (Zhang et al. 2022).

Specifically concerning cortisol, several studies explored its connection with flavonoid treatment. For instance, Zhang and Wang (1997) reported the capacity of naringenin and fisetin to inhibit the activity of the 11 beta‐hydroxysteroid dehydrogenase (HSD‐11β) enzyme, which inactivates cortisol to cortisone (Vantyghem et al. 1994), in guinea pig kidneys, with IC_50_ values of 0.34 and 0.47 mM, respectively. In another study, the authors used oxytetracycline to induce a stress response in silver catfish, leading to increased plasma cortisol levels. The results revealed that co‐treatment with quercetin allowed for the prevention of increased serum levels of cortisol (da Silva Pês et al. 2020). Additionally, implementing in vivo immobilization/swimming‐induced stress Swiss rat models, kaempferol was shown to restore cortisol levels (↓) compared to the stress control group (Habbu et al. 2010). Moreover, two studies have revealed that genistein and daidzein suppress the basal and adrenocorticotropic hormone (ACTH)‐stimulated in vitro secretion of cortisol and corticosterone in porcine adrenocortical cells (Kaminska et al. 2013, 2014). Also, (−)‐epicatechin reduced cortisol resistance in H_2_O_2_‐induced oxidative stress in human monocytes, that is, preserved its anti‐inflammatory activity (Ruijters et al. 2014). Lastly, Ohno et al. (2002) reported that several flavonoids (apigenin, daidzein, genistein, formononetin) were able to significantly decrease the production of cortisol by human adrenal H295R cells in a dose‐dependent manner.

#### 3.3.2 Catecholamines

Numerous catecholamines, including dopamine, epinephrine, norepinephrine, and isoproterenol (a synthetic catecholamine), have been studied concerning their interplay with flavonoids. In this regard, through a variety of processes, including restoring normal levels and modulating their production, or even directly counteracting their action to lessen their effects, flavonoids demonstrate several impacts on the biological effect of catecholamines in a variety of cell types and tissues.

For instance, (−)‐epicatechin ameliorated isoproterenol‐induced myocardial infarction in rats via inhibition of tachycardia, cardiac hypertrophy, and NF‐κB inflammatory signaling pathway (Ponnian 2022). Using a similar model, malvidin was also able to counteract the negative effects of isoproterenol on the cardiac health of rats by restoring defensive antioxidants and ameliorating histopathological changes and impaired mitochondria in the cardiac necrosis (Wei et al. 2017). Furthermore, daidzein restored the impaired dopamine levels in mice with MTPT (1‐methyl‐4‐phenyl‐1,2,3,6‐tetrahydropyridine)‐induced Parkinson's disease (Wu et al. 2022).

Luteolin has been reported to decrease plasma levels of norepinephrine and epinephrine in spontaneously hypertensive rats, as well as to lower the mean arterial pressure and heart rate (Gao, Yu, et al. 2023). Furthermore, epinephrine has been reported to induce lipolysis in rat adipocytes, and quercetin and fisetin were shown to potentiate this effect (Kuppusamy and Das 1994), whereas hesperetin and butein could inhibit it (Kuppusamy and Das 1992). In addition, pinocembrin effectively inhibited collagen/epinephrine‐induced pulmonary thromboembolism in mice while suppressing multiple factors of platelet activation, including aggregation, secretion, and αIIbβ3‐mediated signaling events, in vitro (Li, Liu, et al. 2024). (−)‐Epicatechin could also decrease the maximal platelet aggregation induced by epinephrine in vitro (Sinegre et al. 2019). Furthermore, in a randomized, placebo‐controlled, single‐blind, cross‐over trial of graded cycling to volitional exhaustion with eight males (22.4 ± 3.3 years, BMI: 25.7 ± 2.4 kg m^2^), EGCG decreased epinephrine and norepinephrine plasma levels, measured at different stages of the trial (Churm et al. 2023). Additionally, apigenin ameliorated hypertension in spontaneously hypertensive rats while reducing plasma norepinephrine levels (Gao et al. 2021). Moreover, using an in vivo two‐kidney, one‐clipped (2K1C) hypertensive Sprague–Dawley rat model, genistein was able to alleviate norepinephrine plasma levels, concomitant with an array of kidney‐protective effects, for example, improved renal dysfunction, hypertrophy of the non‐clipped kidney (NCK) and atrophy of the clipped kidney (CK) in 2K1C rats (Poasakate et al. 2022). Lastly, using an in vivo single prolonged stress‐induced PTSD‐like symptoms rat model, myricetin restored norepinephrine levels (↓) in the fear circuit regions, medial prefrontal cortex, hippocampus, and amygdala (Sur and Lee 2022).

#### 3.3.3 Substance P

Similarly to glucocorticoids and catecholamines, Substance P is also affected by flavonoids. These phytochemicals show capacity to impact the biological effect of Substance P on several in vivo models, via restoring normal levels and modulating their production, as well as directly counteracting their action in order to attenuate its effects.

In an in vivo mechanically‐induced hip fracture pain Lewis rat model, pinocembrin was reported to downregulate Substance P levels in the ipsilateral L5 dorsal horn segment (Xing et al. 2022). Moreover, fisetin had the capacity to prevent chronic urticaria‐like symptoms induced by Substance P in mice (Zhang et al. 2023). Additionally, in an in vivo mouse model of high‐fat diet‐induced obesity, apigenin was able to reduce the expression of Substance P in the myenteric ganglia of mice treated with a high‐fat diet (Gentile et al. 2018). Furthermore, naringenin was able to ameliorate loperamide‐induced constipation in mice, concomitant with increased serum levels of Substance P (Yin et al. 2018). Interestingly, two more studies with quercetin have reported similar results. This flavonoid was also capable of enhancing the production levels of Substance P in loperamide‐ or berberine‐induced constipation in murine animals (mice or rats), thus accelerating intestinal peristaltism and gastric discharge (Cui et al. 2024; Liu and Zhi 2021).

## 4 Future Perspectives and Challenges

Life expectancy has risen globally over recent decades, marking one of the greatest achievements of the past century. This trend is expected to continue, and when paired with declining fertility rates, will lead to significant population aging (Proshkina et al. 2020). Between 2015 and 2050, the proportion of people over 60 is projected to nearly double from 12% to 22% (Costa et al. 2025; WHO 2024). However, healthspan—the number of years spent healthy and disease‐free—has not increased at the same pace. Since aging is the main risk factor for most chronic diseases and frailty, addressing population aging has become a major global concern (Proshkina et al. 2020) and discovering new trails to mitigate this has become utterly fundamental. Natural compounds show promise in this regard (Li, Chen, et al. 2024), with phenolic compounds, which encompass flavonoids, being studied for their geroprotective and therapeutic effects on age‐related disorders (Costa et al. 2025). Hence, understanding the precise molecular pathways through which these compounds exert their anti‐stress actions is essential for optimizing their therapeutic potential, particularly in the context of skin health. From our perspective, future research should prioritize the investigation of flavonoids in skin models, especially those related to aging, as current evidence (e.g., PubMed records) highlights this as an underexplored area requiring further advancement before moving to broader applications. Only after establishing this foundation should efforts focus on elucidating how flavonoids mitigate psychological stress and potentially enhance their anti‐aging effects on the skin. This includes examining the mechanisms by which flavonoids interact with stress‐related pathways and downregulate molecular markers of skin aging. To strengthen their stress‐reducing and anti‐aging efficacy, future studies could also explore potential synergistic effects among different flavonoids, combinations with other natural compounds or even conventional therapies, and the design of novel delivery systems that enable efficient skin targeting while minimizing adverse effects. By integrating these approaches—through the development of in vitro, ex vivo, and ultimately in vivo models of stress‐induced skin aging—it will be possible to validate the hypothesis that flavonoids offer a viable strategy for mitigating psychological stress–accelerated skin aging. Nonetheless, despite highly encouraging preclinical outcomes, numerous challenges still hinder their translation into clinical applications. Primarily, flavonoids present low bioavailability, which can be influenced by several factors such as interactions with other nutrients, hepatic metabolism, and modulation by the gut microbiota. Various strategies have been proposed to enhance their bioavailability. For example, formulation modifications—particularly nano‐based delivery systems—have been investigated and demonstrated promising results. However, despite advances in nanotechnology, several barriers persist in translating these systems into clinical use, including regulatory challenges, concerns over long‐term nanoparticle accumulation, and issues related to large‐scale production and reproducibility (Pei et al. 2025). Additionally, structural modification of flavonoids has been considered a valuable alternative, involving chemical alterations such as acetylation, glycosylation, and acylation, among others (Hu et al. 2025). Yet, such modifications result in novel chemical entities with distinct pharmacokinetic and pharmacodynamic profiles that remain insufficiently characterized (Yuan et al. 2024). Another challenge arises from their interaction with gut microbiota; although this relationship has been explored, extending this understanding towards the skin introduces further complexities, such as elucidating the flavonoid‐skin microbiota interplay, which remains largely unexplored. A further concern involves determining appropriate dosing for human use. In many animal studies, the doses employed correspond to unrealistically high and potentially toxic levels when extrapolated to humans. Therefore, establishing standardized dosage parameters in preclinical studies is essential to determine whether the concentrations of extracts or pure compounds used in animal models are both clinically relevant and economically feasible for clinical trials and eventual therapeutic application (Magni et al. 2022). Regarding psychological stress‐related conditions, current clinical data remain insufficient to confirm the therapeutic efficacy of flavonoid‐based formulations, with existing knowledge relying primarily on preclinical research. This lack of clinical evidence underscores the urgent need for human clinical trials. Consequently, future investigations should emphasize mechanistic studies in humans to bridge preclinical findings, particularly in populations affected by stress stemming from environmental (e.g., occupational (work), lifestyle, or social) or genetic factors (Alizadeh et al. 2024; Pannu et al. 2021). Additionally, individual differences—such as ethnicity, sex, age, and socioeconomic background—which are overlooked in preclinical models, may significantly influence outcomes, warranting cautious interpretation of existing preclinical data. A deeper understanding of how flavonoids alleviate the detrimental effects of psychological stress on skin health will, therefore, facilitate the development of innovative interventions aimed at enhancing the overall skin well‐being.

## 5 Conclusions

Nowadays, the fact that psychological stress has nefarious effects on human health is well established, and the negative effects of its mediators, specifically on skin physiology and aging, have been documented in the literature. In general, cortisol and catecholamines, the primary stress mediators, have been related to unfavorable skin outcomes such as reduced collagen and hyaluronic acid production, as well as changes in fibroblast and keratinocyte proliferation, migration, and morphology. Furthermore, several aging mechanisms, including DNA damage, inflammation, cellular senescence, and mitochondrial dysfunction, have been consistently linked to these hormones, albeit mostly concerning other cell types and/or tissues (Duarte et al. 2024). Even though not all case studies confirm these findings, many of them do. From here, it's clear that we need new strategies and solutions to overcome these effects as the cause (stress) appears to be much more challenging to mitigate. The reported biological activities of flavonoids turn these compounds into promising cosmeceutical ingredients. From all identified flavonoids, we could highlight, for example, apigenin, EGCG, or genistein, which were heavily explored throughout the manuscript due to their multifaceted biological potential and can therefore be considered as very promising multifactorial ingredients. In addition, most studies show positive outcomes of flavonoid treatment over the various hallmarks of aging, which could turn them into cellular anti‐aging agents. Nonetheless, more skin‐specific research seems to be required as most studies use other types of cells. On the other hand, flavonoids' positive effects over stress mediators have also been reported, which could assign them a double upstream/downstream protective role, either by conditioning the action of stress mediators or by ameliorating the stress mediators impact in cells, respectively. However, research exploring the potential of these compounds to counteract psychological stress–induced skin aging remains limited, as several challenges and limitations must still be overcome before flavonoids can be fully integrated into clinical practice.

## Author Contributions

Conceptualization: Marco Duarte, Sílvia Santos Pedrosa, P. Raaj Khusial, Ana Raquel Madureira. Writing – original draft: Marco Duarte. Writing – review and editing: Sílvia Santos Pedrosa, P. Raaj Khusial, Ana Raquel Madureira. Supervision: Sílvia Santos Pedrosa, P. Raaj Khusial, Ana Raquel Madureira.

## Funding

This work was supported by national funds from FCT—Fundação Para a Ciência e a Tecnologia, I.P., under the project with DOI: https://doi.org/10.54499/2023.04395.BDANA.

## Conflicts of Interest

Marco Duarte reports financial support was provided by FCT—Fundação Para a Ciência e a Tecnologia. Marco Duarte reports a relationship with Fundação Para a Ciência e a Tecnologia that includes: funding grants. The other authors declare no conflicts of interest.

## Data Availability

Data sharing not applicable to this article as no datasets were generated or analysed during the current study.
